# Evaluation and Cost‐Consequence Analysis of a Community‐Based Digital Exercise Intervention for People With Musculoskeletal Conditions

**DOI:** 10.1002/msc.70142

**Published:** 2025-06-19

**Authors:** Benjamin Wilkins, Maedeh Mansoubi, Jacob Veerapen, Helen Dawes, Benjamin Waller

**Affiliations:** ^1^ Good Boost Wellbeing Bristol UK; ^2^ NIHR Exeter Biomedical Research Centre (BRC) University of Exeter Medical School Exeter UK; ^3^ Finnish Research Centre for High Performance Sport KIHU Jyväskylä Finland

**Keywords:** community remote rehabilitation, complex intervention, digital health, musculoskeletal conditions

## Abstract

**Objectives:**

The objectives of this study are to evaluate the impact and cost‐consequence analysis of a new digital intervention providing water‐ and land‐based exercises for people with musculoskeletal (MSK) conditions.

**Methods:**

Data were collected from May 2021 to December 2023, during which the number of sites providing the intervention increased from 20 to 136. Participant recruitment and characteristics, pain intensity (0–100), physical function (Patient Specific Complaint, 0–100), and health and wellbeing (Office for National Statistics 4, ONS4) were measured. A minimal clinically important detectable (MCID) change of 15% was used. Symptoms, function and wellbeing were measured at 6, 12 and 26 weeks. A cost‐consequence analysis was conducted comparing 12 digital exercise sessions to 6 face‐to‐face (F2F) physiotherapy sessions.

**Results:**

In total, 4429 participants with MSK conditions, who completed at least 1 exercise session, were included in this study. 3515 (79.4%) were female, average age 58.7 ± 15.3 years old, 13% registered as ethnicity other than white, 33.5% were in the third quartile for high deprivation and 44.2% were sedentary. The knee (33.3%) was the most affected body region. In total, 40,995 exercise sessions were completed (91.6% water‐based), and the average sessions per user were 9.3. Small significant (*p* < 0.05) improvements in function, happiness, and anxiety were seen at 6 weeks, with improvement in function and anxiety maintained at 12‐ and 26‐week follow‐ups. At 6 and 12 weeks, 33.8% and 38.6% reached MCID in pain intensity and 40% and 45% in physical function, improvements which are similar when compared to expected outcome of face‐to‐face physiotherapy. Cost‐consequence analysis indicated an estimated saving of £168.72 per participant compared to F2F physiotherapy.

**Conclusion:**

This digital MSK exercise solution delivered to people with MSK conditions had a positive effect on pain intensity and physical function with considerable potential cost savings.

## 1 Introduction

National Health Service (NHS) data highlights that waiting lists for musculoskeletal (MSK) services in the UK are currently 14 weeks, with significant pressure to improve efficiency of MSK services (Cottrell and Russell 2020). To reduce this burden, there is considerable focus on developing new technology‐enabled health care with the potential to expand the capacity of primary care, to provide more integrated care, and to offer support to patients with self‐management and independent living (Kraef et al. 2020; Young and Nesbitt 2017). Digital services developed with healthcare professionals have the potential to provide wider access to MSK services, but to date, evidence on the effectiveness, cost and cost‐effectiveness of such technologies is limited, eventhough these have been identified as important areas for research focus (Fandim et al. 2023). For example, in two recent studies, mobile applications appear to have been built to a sufficient quality, that is, good user experience and based on scientific evidence, but with limited effect on symptoms, physical activity and behavioural change in people with chronic long‐term health conditions (Bricca et al. 2022; Zangger et al. 2023).

Digital solutions can be scaled to a wider population quickly and at low cost, especially where delivery does not require a skilled healthcare professional. Integrating digital solutions into the delivery of MSK services may also improve access to a beneficial service, regardless of ethnicity, geographical location or physical mobility, allowing regular access to evidenced, tailored progressive interventions (Cottrell and Russell 2020; Merolli, Gray, Choo, Lawford, and Hinman, 2022). For example, a pilot feasibility study indicated that it is safe and feasible for older adults to participate in a home‐based digital MSK programme, highlighting the potential of digital solutions for this population (Daly et al. 2021). However, to date, the scope of delivery options has been limited mainly to home environments, and different approaches, such as the implementation of digital solutions in existing community spaces. for example, leisure centres, has not been evaluated (Areias et al. 2023). A potential digital solution developed which delivers bespoke exercise programs for people with MSK conditions in community leisure centres and at home and its feasibility for a single site has been published (Wilson et al. 2024). Such digital based intervention options need sufficient evidence, including efficacy, safety and adherence to digital security, to support their wider implementation.

Digital solutions are, by nature, complex systems; therefore, commonly used efficacy and effectiveness study designs are not always suitable for the evaluation of complex interventions, especially those under development and constantly changing (Skivington et al. 2021). As a digital solution scales from a few delivery sites to multiple sites in different communities, the delivery changes according to the environment, for example, pool depth, personnel, for example, skilled or unskilled healthcare professionals, participants, for example, heterogeneous for age, condition(s), severity etc. In addition to effectiveness and efficacy, evaluation of the implementation of a scaling intervention requires understanding who is engaging with the intervention, and the efficiency, that is, cost‐consequence, of digital solutions should be considered as a key assessment criterion (Murray et al. 2016). Engagement and adherence to a digital programme reveal insights into different demographic and condition‐based groups' ability to participate in MSK exercise prescription. Understanding how various age brackets, ethnicities, cultures, geographies and MSK conditions engage with the programme aids in tailoring interventions for better inclusivity (Giesbrecht and Crooks 2016) and can form part of the ongoing development of a complex intervention (Skivington et al. 2021). Demographic considerations, such as age, physical limitations, and location diversity, influence the acceptance and accessibility of such interventions (Mastellos et al. 2016).

The objectives of this study are to evaluate the impact and cost‐consequence analysis of a new digital intervention for people with MSK conditions as it scales from a few controlled delivery sites to many delivery sites nationally in the United Kingdom.

## 2 Methods

### 2.1 Study Design

This is a quantitative evaluation of a digital service for MSK conditions during its roll out from 20 controlled delivery sites to 136 sites across the United Kingdom over a 2‐year period. It is reported following the STROBE guidelines (Cuschieri 2019; von Elm et al. 2014). The feasibility and implementation of the digital intervention has been described previously (Wilson et al. 2024).

### 2.2 Ethical Statement

The University of Exeter ethical board approved this secondary data analysis after evaluating the intervention process for consent and the information that participants were provided. Written consent was signed by participants through the digital tablet or phone‐based application (Good Boost Wellbeing, UK), which included consent for inclusion of anonymised data in service evaluation and research purposes. The process adheres to the General Data Protection Regulations (GDPR) and is stored according to good research practice and data protection. The application is used for data collection and exercise delivery and meets the standards for digital healthcare interventions and is approved by the Organisation for the Review of Care and Health Apps (ORCHA), Digital technology Assessment Criteria (DTAC) and National Health Service standards.

### 2.3 Patient and Public Involvement Statement

From the ideation of the first iteration, older adults with MSK conditions and consumers, that is, community venues, health care professionals, charity and leisure industry stakeholders, were involved in the intervention development. The development of the evaluation involved a wider range of stakeholders including the community venues and their operators, clinical commissioning groups, non‐governmental organisations with an interest in health and wellbeing, and the leisure industry and digital health registration organisations. This was conducted through different methods, including but not exclusively, focus‐groups, interviews, user‐centred design, and app feedback systems. People with MSK conditions and experts were recruited through national MSK charity (e.g., Arthritis Action, vs. Arthritis and National Axial Spondyloarthritis Society).

### 2.4 Participant Recruitment and Inclusion

This study is an evaluation of data collected through routine delivery of the solution; therefore, participants voluntarily participated in and self‐funded the intervention as part of the management of their MSK condition. Participants could attend as many or as few sessions as they wished. No time duration or attendance limitation was placed on them. Inclusion criteria were (1) completed at least one exercise session within the digital ecosystem irrespective of context, this required a time stamp for starting and finishing the session within the expected duration of the chosen session, type or environment, (2) interaction between 1st May 2021 and 31st December 2023, (3) agreed to terms and conditions, data sharing and research consent on registration, (4) aged over 18 years old and (5) resident in United Kingdom. Exclusion criteria were (1) partially completed sessions (impossible to know if participant actually completed an exercise) (2) participant was associated with an external testing protocol or evaluation (e.g., Apple, Google, ORCHA) (3) participant is a facilitator at a private or community venue, (4) suspicion that users profile had been shared to one or more user either (i) had completed simultaneously two or more sessions or (ii) participant had completed more than 4 session on at least 2 separate occasions (5) duplicate account based on name, year of birth, gender and postcode, in which case most used account was kept.

### 2.5 Intervention

**FIGURE 1:**
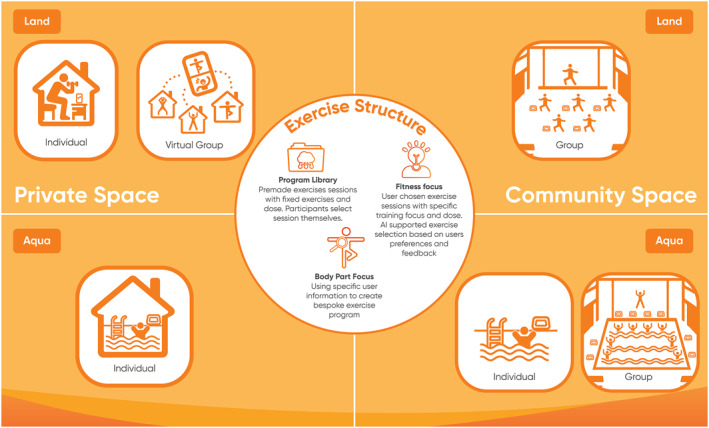
The intervention structure implemented in the two different training contexts (land and aquatic). two different environments (community and private spaces) and in group and individual delivery.

The system delivers bespoke exercise programs with the aim of impacting the participants' symptoms and functional limitations while considering possible MSK and other medical conditions. Participants can access the exercise programs via their phone/tablet at home or on a tablet in a community setting (usually a leisure venue) with exercises being completed on land or in water (Figure 1). The sessions are delivered in a group with or without a facilitator. Facilitators for community and private groups are provided with an approved online training programme but are not required to be qualified healthcare professionals. The facilitators' role is to promote the community and social aspects of the groups while the digital solution provides the exercise programs adapted for the participants.

The participant can choose between three different types of exercise programs; (1) premade fixed exercise sessions which focus on specific body part, training type and duration, for example, breathing exercises. (2) Exercises sessions with specific exercise physiological impact, that is, strength, aerobic fitness, balance, core stability and flexibility, and allows user to select intensity, body area to be impacted, training position (e.g., sitting, standing), session duration and equipment. (3) A multimodal joint focused session (containing strength, aerobic, flexibility and balance exercises) based on data collected from the participant at the initial registration and intensity. Difficulty of exercises is tailored to the participants symptoms and functional limitations. Additionally, the participant can influence the session duration, training position, for example, sitting standing or lying and the selection of equipment to be used in the session. It is important to note that baseline health data, for example, MSK condition and symptoms, were only collected from the participants choosing this third training option. Options (1) and (2) lower the threshold to access, while option (3) provides more specificity but the interaction with the application is more demanding for the participant through increased questioning. Session options (2) and (3) are also adapted using data from the participants' feedback on previous session's intensity, liked/disliked exercise, levels of fatigue and changes in symptoms, creating a constant refinement of the exercise sessions provided. The intensity and difficulty level of the exercises were also adapted to reported comorbidities such as cardiac and respiratory conditions, the severity of the associated symptoms, and their impact on the participants' physical functioning. The exercises available and session structures have been developed in collaboration with multiple experienced clinicians and stakeholder groups and have been refined through user testing (see previous section).

### 2.6 Baseline Demographics

All participants provided baseline physical activity prior to starting the intervention using the International Physical Activity Questionnaire (iPAQ) (Lee et al. 2011). Health and wellness were initially measured with the WHO EQ5D Comorbidities were collected through an optional health questionnaire (Feng et al. 2021) but were replaced early in the intervention delivery with the five dimensions of health and Office for National Statistics four questions for personal wellbeing (ONS4) (Tinkler 2015). This change was in response to the request by the leisure industry and use of the ONS4 started from May 2023 through the data collection. Therefore, data from the EQ5D are presented only in the supporting data. The ONS4 questionnaire consists of four domains: life satisfaction, worthwhileness, happiness and anxiety using a numerical rating scale (NRS) 0–10 with ‘not all’ = 0 and ‘completely’ = 10. For life satisfaction, worthwhileness and happiness, a higher score is better, while anxiety is reversed with a lower score indicating less anxiety.

Those participants participating in the bespoke exercise intervention (option 3 above) were asked about affected joint, diagnosis, duration of symptoms, symptoms measured with average and max pain intensity (visual analogue scale (VAS) 0–100: 100 = worse pain intensity) (Bond and Pilowsky 1966). Physical function was measured using the participant‐specific compliant (PSC), which requires the participant to grade (VAS 0–100: 100 = extreme difficulty) the difficulty they have with a specific movement which is important to them, and they were asked to grade their difficulty with that specific movement at the follow‐up time points (Mathis et al. 2019). Comorbidities were collected through an optional health questionnaire.

### 2.7 Outcomes

The outcomes for the evaluation were developed through an iterative process with multiple stakeholders with each having their own outcome of importance; therefore, the authors have not chosen a primary outcome. The data was collected through the three different user facing applications (HUB Good Boost Wellbeing, UK, v1.0.1–2.0.22, MT Good Boost Wellbeing, UK, v 1.0.4–1.0.7, AM Good Boost Wellbeing UK, v1.0.1–1.0.3). The HUB delivers the service in the community and settings and MT and AM deliver the service in the private setting.

### 2.8 Outcomes Measures

MSK condition‐specific metrics were asked from all participants reporting at baseline a specific body part at 6, 12 and 26 weeks. Data was collected at 52 weeks as well, but not used in this analysis as few participants would have reached this time point at data extraction. Outcomes are average and max pain intensity, participant specific complaint, global improvement in body part (seven‐point Likert from very worse to completely better) and perceived benefit from the exercises for that body part 0–100 (0 = no benefit, 100 = complete benefit). During piloting, it was observed that a few participants would not truthfully answer the follow‐up questions due to time restrictions; therefore, response to follow‐up MSK outcomes was optional and users could choose not to answer these. Further, follow‐up questions only appeared in the application during a 3‐week window around the follow‐up time and would appear on each log in to the application until completed or the time window expired. The outcomes were synchronised across the three different applications.

### 2.9 Economical Evaluation

The economic viability of the intervention will be assessed using a direct cost‐comparison between the delivery of 6 physiotherapy sessions delivered in the local outpatient service with 12 sessions in a group class or remotely in a private setting (Hannink et al. 2022). Classes will not be differentiated for context, that is, land or aquatic, but will be differentiated for environment, that is, delivery in a community versus private setting. Assumptions are based on a full class of 10 participants, where the community venue space cost is £30 per hour, facilitator for private and community setting are a similar cost of £15.00 + 20% for overheads = £18.00, yearly cost of 10 sets of hardware and software is £4,000, each venues delivers 2 sessions of 10 participants as week, for 44 weeks of a calendar year, monthly subscription cost of the virtual group application is £5 per calendar month. 30 km travel per session for the participant was used (Hannink et al. 2022).

### 2.10 Adverse Events

Adverse event reporting was through a direct email and focus group meetings. Facilitators could similarly send direct feedback on behalf of the participant through interviews, email or feedback systems within the community application.

### 2.11 Statistical Analysis

Statistical analysis was performed using IBM SPSS Statistics software version 28.0 for Windows. Descriptive statistics were performed for demographics and baseline scores and expressed as mean and standard deviation, while nominal data were described in frequencies and percentages. Minimal clinically important difference (MCID) of 15% for pain intensity and physical functioning (PSC) was reported as frequency (Salaffi et al. 2004). Complete representation data will be presented in the Supporting Information S1. Missing data and loss to follow‐up, that is, did not complete the next follow‐up outcomes, was expected due to the nature of data collection (participant was free to choose when they attend). Population sizes and the amount of missing data will be presented for transparency.

Were participants registered more than one affected body part, the most severely affected body part, based on pain and function, was chosen for follow‐up evaluation. An independent 2‐tailed *t*‐test with an alpha of 0.05 was used to compare pain intensity (max and average), function (PSC) and all 4 domains of the ONS4 questionnaire at the different time points of 6, 12 and 26 weeks. Additionally, the ONS4 NRS scales were grouped into 4 categories. Mean change in outcomes is reported with 95% confidence intervals (CI 95%). The McNemar's test, for paired data, was used to compare the results of categorical data for changes in overall categories of the ONS4 categories; again, a *p* value below 0.05 was considered significant.

## 3 Results

**FIGURE 2:**
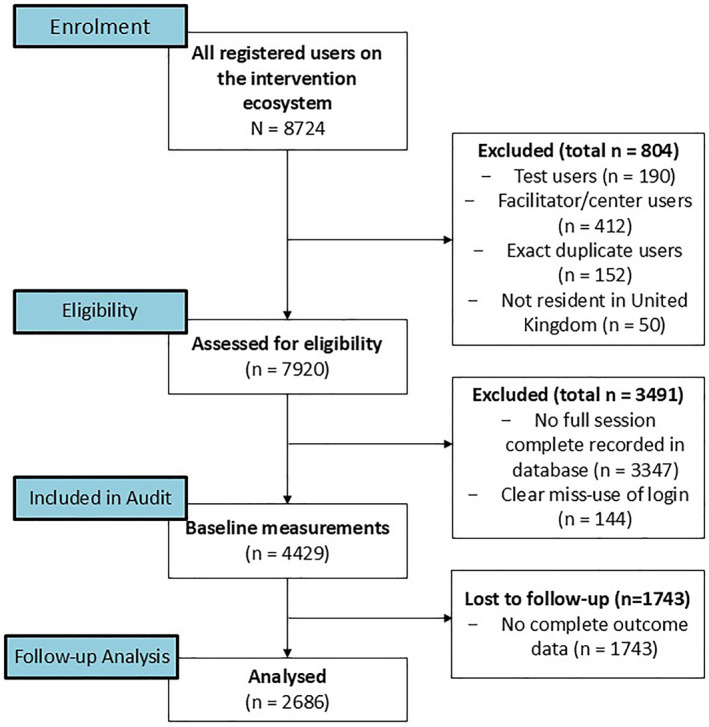
Flow chart representing the recruitment into this study.

Figure 2 shows the recruitment process for this evaluation and Table 1 shows the baseline demographics of the included participants.

**TABLE 1:** Describes the included participants.

Baseline demographics (*N* = 4429)	
Female	3515 (79)
Age, mean (SD)	58.7 (15)
Ethnicity	
White	3398 (87)
Black	161 (4)
Asian	147 (4)
Mixed ethnicity	51 (1)
Other ethnic group	41 (1)
Prefer not to say	87 (2)
Unknown	24 (1)
Social‐economic deprivation	
Low	1208 (35)
Moderate	1085 (32)
High	1153 (33)
Distance to venue	
< 5 km	2373 (54)
5–10 km	790 (18)
5 km	556 (13)
Employment	
Retired	1902 (43)
Employed	651 (15)
Employed–part time	564 (13)
Unable to work	474 (11)
Prefer not to say	171 (4)
Student	42 (1)
Missing	625 (14)
Recommendation pathway	
Leisure venue	1445 (33)
GP/Physio	1043 (24)
Word of mouth	534 (12)
Other	375 (8)
Social prescriber	186 (4)
Poster/leaflet	98 (2)
Missing	229 (5)
Not asked	519 (12)
Physical activity (iPAQ)	
Low	1434 (44)
Moderate	1287 (40)
High	525 (16)

Medical history	
No. affected joints, mean	1.5
All affected locations (*n* = 5605)	
Shoulder	648 (12)
Lower back	1258 (22)
Hip	886 (16)
Knee	1468 (26)
Generalised pain	1345 (24)
Primary affected location (*n* = 3608)	
Shoulder	253 (7)
Lower back	758 (21)
Hip	505 (14)
Knee	1190 (33)
Generalised pain intensity	902 (25)
MSK conditions (*n* = 3208)^a^	
Conditions specified (*n*)	37
Osteoarthritis	942 (29)
Fibromyalgia	178 (6)
Inflammatory Arthritis	173 (5)
No diagnosis	740 (23)
Post‐surgery (*n*)^a^	400 (9)
Surgery types specified (*n*)	14
Knee arthroplasty	164 (41)
Hip arthroplasty	77 (19)
Chronicity^a^	
Acute (0–12 weeks)	702 (19)
Sub‐acute (12–24 weeks)	238 (7)
Chronic (> 24 weeks)	2668 (74)
Max pain intensity^a^, mean (SD)	47.6 (24.1)
Average pain intensity^a^, mean (SD)	44.5 (22.9)
Physical function (PSC)^a^, mean (SD)	65.4 (25.2)
Comorbidity (*n* = 3110)^b^	2443 (79)
Other musculoskeletal	1949 (64)
Diabetes	388 (12)
Respiratory	545 (18)
Cardiac	872 (28)
Neurological	323 (10)
Renal	279 (9)
Cancer (active)	43 (1)
Cancer (recovery)	205 (7)

^a^ Primary complaint.

^b^ Responders to medical questions.

### 3.1 What Had Been Delivered?

**FIGURE 3:**
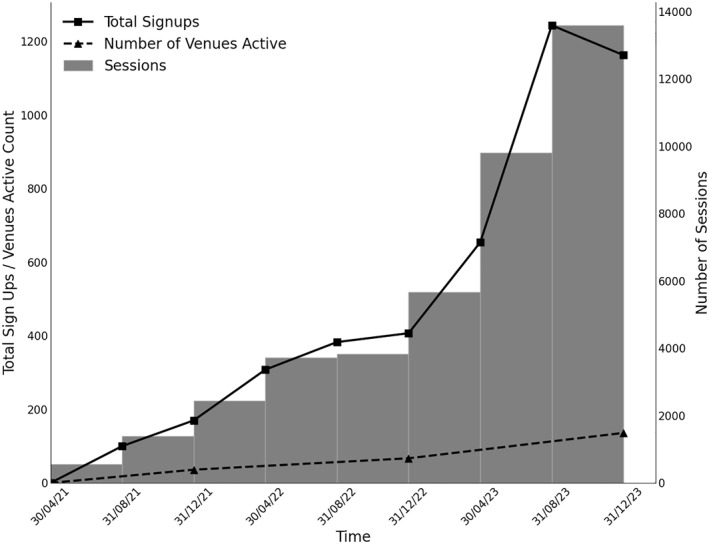
Graph showing the instantaneous uptake of new participants and session completed over time.

Over the 30 months, there was steady growth in participation with an acceleration in uptake during 2023. In May‐December 2021, 270 participants (6.71%) registered, followed by 1098 (27.3%) in 2022 and 3061 (75.8%) in 2023. During this period, a total of 40,995 exercise sessions were completed at an average of 9.2 sessions per participant. Of these sessions, 36,973 (90%) were completed in a community centre, with 2437 (6.6%) land‐based and 34,536 (93.4%) water‐based sessions. Figure 3 shows the instantaneous uptake of new participants and sessions completed over the development phase. As new sign‐ups increase after September 2022, the number of sessions appears to increase at a greater rate, potentially indicating that users were returning to complete additional sessions more frequently. 1449 aquatic and 972 land‐based sessions were completed in a private setting. Out of the total sessions, 3453 (8.4%) were land‐based, and 37542 (91.6%) were water‐based. Water‐based exercise was the first session for 3930 (88.7%) participants. The number of sites delivering the intervention increased over the data collection period from 36, 67 and 136 venues in 2021, 2022 and 2023, respectively.

### 3.2 Interaction of Implementation With Outcomes

In the whole population, there was no change in average or max pain intensity reported at any time point when broken down to either primary body part or per body part. However, a significant decrease in difficulty in physical function was seen as early as 6 weeks. Difficulty in physical functioning (PSC) decreased significantly with mean changes of −10.86 (−12.59, −9.12, *p* < 0.000), −10.03 (−12.05, −8.02, *p* < 0.000) and −10.00 (−13.16, −6.84, *p* < 0.000) at 6‐, 12‐ and 26‐week follow‐up, respectively. No effect on pain intensity (max or average) was seen (Table 2).

**TABLE 2:** Changes in pain and function over time.

	*n*	Mean	SD	Mean change (95% CI)	*p* value
Max pain (0–100)					
Baseline	1671	47.57	24.10	—	—
6‐week	1600	46.50	23.16	−1.07 (−2.69, 0.55)	0.098
12‐week	826	47.79	23.06	0.23 (−1.73, 2.18)	0.588
26‐week	273	49.41	23.43	1.85 (−1.17, 4.86)	0.880
Average pain (0–100)					
Baseline	1671	44.49	22.94	—	—
6‐week	1597	45.06	23.27	0.57 (−1.02, 2.15)	0.758
12‐week	825	46.33	22.32	1.83 (−0.05, 3.71)	0.971
26‐week	273	47.27	23.87	2.78 (−0.27, 5.82)	0.967
Function (PSC) (0–100)					
Baseline	1671	65.35	24.25	—	—
6‐week	1600	54.50	25.31	−10.86 (−12.59, −9.12)	0.000
12‐week	826	55.32	23.62	−10.03 (−12.05, −8.02)	0.000
26‐week	273	55.36	24.56	−10.00 (−13.16, −6.84)	0.000

**FIGURE 4:**
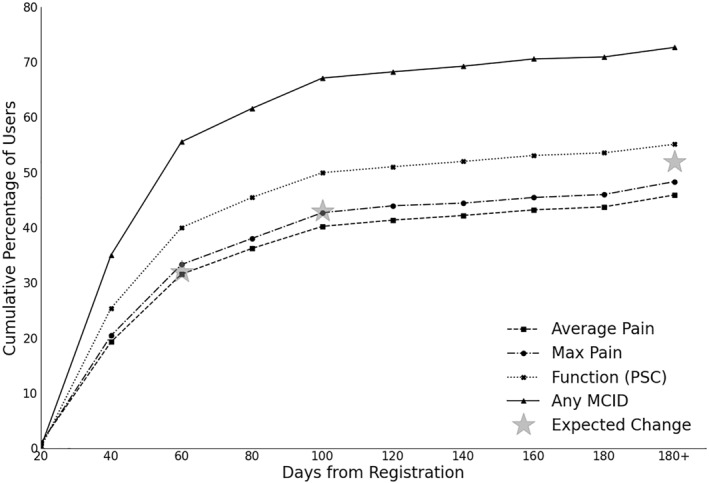
Participants reaching minimal clinical important difference (MCID) for pain intensity or function (PSC) or any outcome over time.

In total, at 6 and 12 weeks 33.8% and 38.6% reached a minimal important detectable change (MCID) of 15% change in pain intensity and 40% and 45% of participants for physical function (PSC) (Figure 4). Compared to previous data (De Vos Andersen et al. 2017), 6‐week follow‐up improvements in MCID pain intensity and physical function were equivalent compared to face‐to‐face physiotherapy. MCID in either pain intensity or physical function was reached by 56.5% and 67.8% of participants at 6 and 12 weeks, respectively. Global change results indicate that at 6 weeks after starting the intervention, 65% reported at least a slight to large improvement in their primary condition (see Supporting Information S1).

### 3.3 Health and Wellness

Already at 6 weeks of participation, a small but significant improvement in life happiness, worthwhileness and anxiety was observed (Table 3). Improvements in anxiety were still significant at 12 weeks −0.43 (−0.68, −0.18 *p* = 0.001). There was also a significant decrease, 40.60%–33.15% in participants categorised into the high anxiety category (*p* < 0.001) at 6‐week and maintained at longer follow‐up (see Supporting Information S1).

**TABLE 3:** ONS4 (Describe better).

	*n*	Mean	SD	Mean change (95% CI)	*p* value
Life satisfaction (0–100)					
Baseline	2931	6.66	2.25		—
6‐week	1151	6.88	1.87	0.22 (0.09, 0.36)	0.001
12‐week	550	7.08	1.81	0.43 (0.25, 0.60)	0.000
26‐week	138	6.96	1.86	0.31 (−0.02, 0.63)	0.059
Worthwhileness (0–100)					
Baseline	2931	7.33	2.16		—
6‐week	1151	7.40	1.89	0.08 (−0.06, 0.21)	0.141
12‐week	550	7.54	1.82	0.21 (0.04, 0.38)	0.016
26‐week	138	7.63	1.83	0.31 (−0.01, 0.62)	0.052
Happiness (0–100)					
Baseline	2931	6.96	2.34		—
6‐week	1151	7.23	2.04	0.26 (0.12, 0.41)	0.000
12‐week	550	7.02.00	2.02	0.24 (0.05, 0.43)	0.013
26‐week	138	7.19	2.26	0.23 (−0.16, 0.61)	0.134
Anxiety (0–100)^a^					
Baseline	2931	4.56	2.98		—
6‐week	1151	4.26	2.69	−0.30 (−0.49, −0.11)	0.002
12‐week	550	4.13	2.71	−0.43 (−0.68, −0.18)	0.001
26‐week	138	4.36	2.90	−0.20 (−0.70, 0.30)	0.222

^a^ Reverse score, that is, lower is better.

### 3.4 Participant Satisfaction

Through co‐design with stakeholders and users, using an iterative approach to the intervention the star rating (out of 5) the average increased from 2.2 in 2021 to 3.8 in 2022 and 4.5 in 2023. In 2023, 90.5% of sessions received a rating of 4 or 5 stars compared to 10% in 2021.

### 3.5 Cost Evaluation

A summary of the calculations and results can be found in Table 3. When including all costs for travel between 6 sessions with a physiotherapist and 12 intervention sessions in a community venue with facilitator, the intervention created a saving of £168,72 per participant (Table 4).

**TABLE 4:** Cost calculation for cost evaluation.

Intervention delivery	Cost	Benefit
Physiotherapist in clinic	Physiotherapist band 6 £52.00* Travel £12.60 Carbon footprint £0.48	Face‐to‐face interaction preferred by majority of patients (recent Oxon survey) and allows for complex management
	Cost per session = £65.08 Cost per full prescription @ 6 sessions = £390.48	
	No cost or payment by participant	
Digital‐supported intervention in community venue in class	Pool/studio cost = £3 Facilitator = £1.80 Technology = £0.60 Travel £12.60 Carbon footprint £0.48	No limit on the number of classes to be delivered Technology could be used to deliver individual sessions
	Cost per session = £18.48 Cost per full prescription @ 12 sessions = £221.76	No waiting lists and can be accessed immediately
	Cost on point of contact to participant is on average £5	
Digital‐supported intervention in private. Venue in virtual group at home	Facilitator = £1.80 Technology = £0.63 Travel £0.00 Carbon footprint £0.00	No limit on the number of classes to be delivered Technology could be used to deliver individual sessions
	Cost per session = £2.43 Cost per full prescription @ 12 sessions = £29.16	No waiting lists and can be accessed immediately
	Cost on of technology can be paid by group provider (charity) or the participant	No travel costs required
		Size of virtual groups can be unlimited to 2–3 times number of community venue classes

### 3.6 Adverse Effects

In total, 9 participants reported stopping participation due to increased pain intensity following a session, and 5 reported an increase in pain intensity but did not stop participation, one person assisting a participant had a stroke in the pool. Three participants complained about the sensitive nature of the questioning.

## 4 Discussion

To the best of our knowledge, this is the first study to present the evaluation of a community‐based digital intervention implementation in a real‐world situation. The intervention had a positive impact on physical function, life satisfaction, life worthwhileness, and anxiety. Additionally, an equivalent effect on pain was seen as expected from face‐to‐face physiotherapy treatment in an outpatient setting at short‐ and long‐term follow‐up (De Vos Andersen et al. 2017), but at a third of the cost. Further, the interventions' impact on pain intensity, physical function, wellness and anxiety is in line with a recent study on the impact of mhealth apps on people with chronic or multi‐morbidity (Zangger et al. 2023) and MSK conditions (Hong et al. 2022; Wang et al. 2023). Our evaluation revealed increasing engagement with the programme over time, indicating satisfaction and acceptance with the intervention.

This study includes data from 4429 participants with MSK conditions who have self‐selected to engage in the intervention, creating a heterogenous population with a wide range of MSK conditions and co‐morbidities, baseline pain intensity ranging from 0–100, affected body and chronicity. The heterogeneity in participants may also explain the lack of measurable effect of the intervention on absolute pain intensity scores; however, the numbers reaching MCID are equivalent. The cohort consisted of a higher proportion of females with a slightly higher ethnicity of white (87%) than the United Kingdom population (82%). However, even though the intervention was also self‐funded by the participation (between £3‐£8 per session), there was an equal distribution between low, moderate and high areas of socioeconomic deprivation, indicating that people are prepared to pay for self‐management of their MSK conditions. Our study addresses a highlighted lack of research examining programmes tailored to suit the individual's health condition, and our findings support the positive benefit of refining, and co‐designing interventions, and an overall willingness of individuals to invest in self‐managing MSK conditions (Rowley et al. 2018).

Notability, at registration, 44% of participants had high levels of anxiety, as measured with the ONS4 and a higher level of functional difficulty than pain, as measured with the PSC. The link between kinesiophobia, anxiety and functional capacity in people with chronic MSK conditions has been well documented (Burston et al. 2018; Grande et al. 2025). This could partly explain why the participants chose the aquatic exercise training over the land‐based alternative. Additionally, our results indicate that there was a decrease in anxiety and improvement in functioning at both 6 and 12 weeks, which could be related to increased confidence, which has been previously reported (Fisken et al. 2012). Additionally, training in a group setting has been reported as providing additional peer‐support (Wilson et al., 2024), which can lower barriers to exercising (Grande et al. 2025). Future research should focus on investigating these effects on objectively measured function and changes in overall physical activity behaviour.

Exercise, on land or in water, is a long‐accepted cornerstone in the management of people with musculoskeletal conditions (Fransen et al. 2015; Heywood et al. 2017); however, delivery through applications available on the App Store is of varying quality and does not seem to stimulate behavioural change towards physical activity (Bricca et al. 2022; Zangger et al. 2023). The results indicate that 62% of participants completed 5 or less sessions, which could be in part related to the challenging environment for delivering the intervention, that is, leisure centre with poor internet connection (Wilson et al. 2024). It must also be noted that date extraction was conducted at a point where new participant numbers were constantly increasing and many would not have had the opportunity to attend many sessions. On average, individuals engaged in 10 sessions, which may be due to the use of facilitators to support delivery where digital literacy may otherwise have been a barrier, and exercising in a group may facilitate social interaction, thus augmenting the impact of exercise (O’Keeffe et al. 2017). Therefore, once the initial barriers to participating in community‐based exercise groups have been overcome, long‐term adherence to exercise is possible. The high self‐selection of aquatic exercise (92%) indicates the potential for this service to attract participants who would not attend land‐based sessions or find aquatic exercise a lower threshold for initiating exercise (Dobson et al. 2016).

In a recent Delphi study, clinical effectiveness and cost‐effectiveness were identified as priorities in digital health and MSK related pain intensity. The intervention in this study is an agile complex intervention undergoing constant iterations and as such the methods used to evaluate its impact, while not an RCT study, are in line with the methodological approach presented in the updated MRC guidelines (Skivington et al. 2021). NHS England data estimates the annual cost of those with one long‐term condition to be £3000, which almost triples for those with two or three long‐term conditions. Long‐term and multiple long‐term conditions cost health and social care an estimated £115.2 billion annually, the results of this study indicate that it is an efficient intervention given the potential savings per user and impact on MSK related outcomes. Cost evaluation indicates a considerable saving potential through reduced cost of delivery with similar outcomes.

The recruitment and inclusion, while allowing for a heterogenous population for this study and demonstrating the diverse population of people who would use such an intervention, was based on self‐selection (Nohr and Liew 2018) and saw a high loss of participants to follow‐up as expected with such an intervention (Biele et al. 2019). Therefore, the result of this study is open to bias and possible over estimation of effect. The high number of participants registering in the app but not completing a session indicates a high interest in the self‐management digital applications; however, the reasons for this churn are currently unknown and require further investigation.

## 5 Conclusion

This study indicates that digital exercise for people with MSK conditions, especially aquatic therapy, is safe, acceptable, implementable, scalable, and transferable across different contexts and has the potential to be cost‐effective. As with all digital solutions, the intervention is a complex adaptable system, which will constantly improve beyond the publishing of this evaluation.

## Author Contributions

Guarantor: H.D. Conception and design: primarily B.Wi., all authors. Acquisition of the data: B.Wa. and J.V. Analysis and interpretation: all authors. Draughting. Critical revision and final approval of the article: all authors. Statistical expertise: B.Wa., H.D., M.M. and J.V. Collection and assembly of data: B.Wa., H.D., M.M. and J.V. All authors agree to be accountable for all aspects of the work in ensuring that questions related to the accuracy or integrity of any part of the work are appropriately investigated and resolved: all authors.

## Consent

Consent for participation in the study was obtained from all the participants. No additional identifiable data is published in the manuscript.

## Conflicts of Interest

Benjamin Waller, Jacob Veerapen and Benjamin Wilkins were employed by Good Boost Wellbeing at the time of data collection and reporting.

## Supporting information

Supporting Information S1

## Data Availability

All data relevant to the study are included in the article or uploaded as supplementary information as aggregated data. It is not possible due to legal limitations to share the individualised data.
