# Supplementary material for: Evaluation and Cost‐Consequence Analysis of a Community‐Based Digital Exercise Intervention for People With Musculoskeletal Conditions
Source: Musculoskeletal Care. 2025 Jun 19;23(2):e70142. doi: 10.1002/msc.70142 (PMC12178840; doi:10.1002/msc.70142)
Supplement: Supplementary file 1 — Supporting Information S1 [file MSC-23-e70142-s001.docx]

Implementation Evaluation

*All data refers to UK based participants who signed up between 1^st^ May 2021 and 31^st^ December 2023 and completed at least one exercise session within those dates. Uncompleted exercises sessions are not counted, they void the assumption that the user had actually completed enough of the content of the exercise session to be attributed to a meaning exercise interaction with the intervention.*

*Participant accounts have been removed which appear to have been used by multiple users which is described as having completed multiple sessions on a single day on more than 4 occasions (the suspicion being that a participant has shared their account).*

This appendix is structure in line with the evaluation planned described in the main manuscript and in the below figure 1:


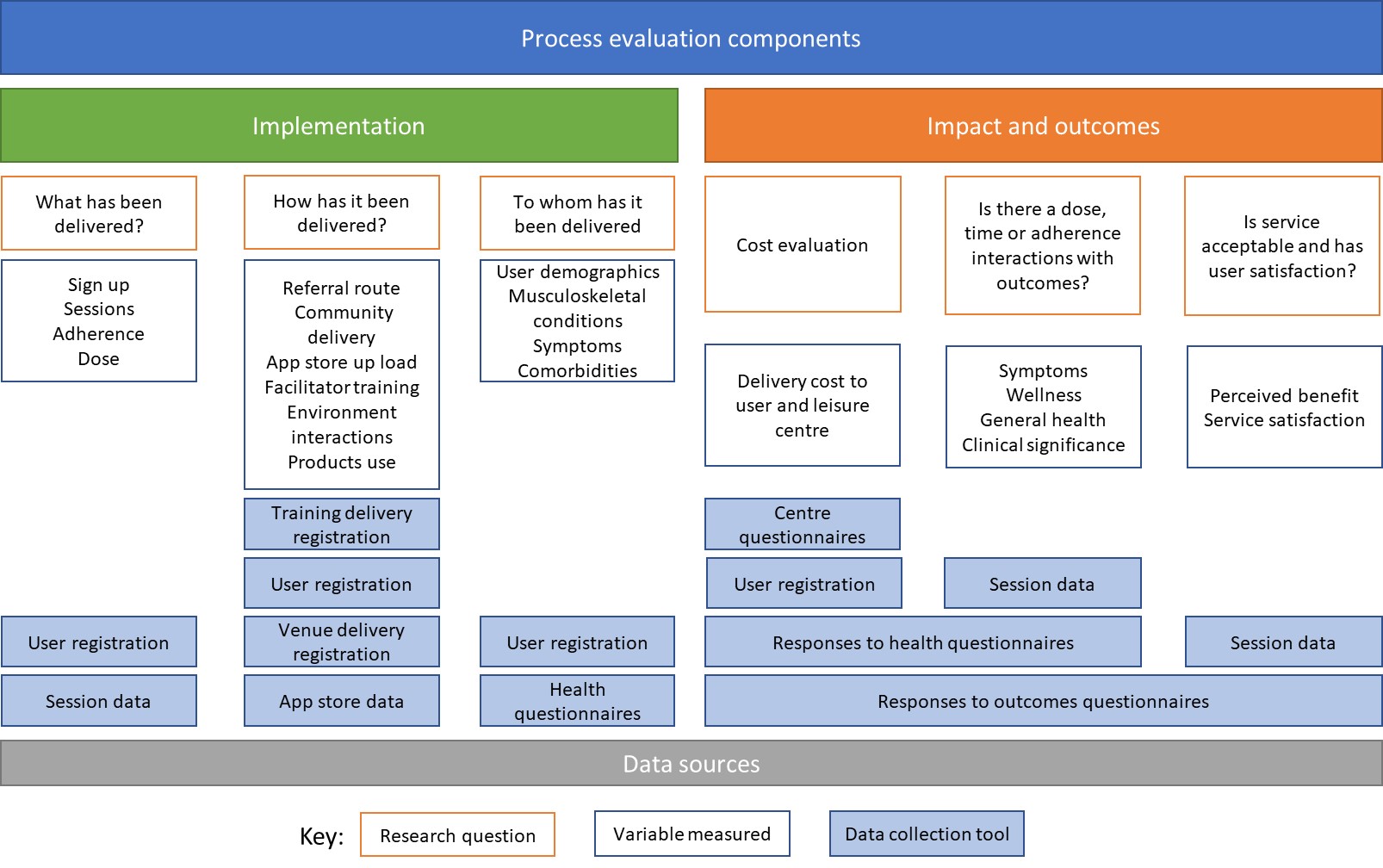


Figure 1 Evaluation approach for the clinical audit

Contents

[Table of Figures ii](#_Toc198372413)

[Table of Tables iii](#_Toc198372414)

[S1 Implementation Evaluation 1](#_Toc198372415)

[1.1 What has been delivered? 1](#_Toc198372416)

[1.1.1 Participant registration per environment 1](#_Toc198372417)

[1.1.2 Sessions per environment 1](#_Toc198372418)

[1.1.3 Adherence 2](#_Toc198372419)

[1.2 How has it been delivered? 2](#_Toc198372420)

[1.2.1 How did you hear about the service? 2](#_Toc198372421)

[1.2.2 Venue recruitment (Community) 3](#_Toc198372422)

[1.2.3 Number of facilitators trained 4](#_Toc198372423)

[1.2.4 Location hot spots for uptake 4](#_Toc198372424)

[1.2.5 Location - Distance to Registration Venue (Community) 5](#_Toc198372425)

[1.2.6 Private (Home) intervention uptake 5](#_Toc198372426)

[1.3 To Whom has it been delivered? 6](#_Toc198372427)

[1.3.1 Demographics 6](#_Toc198372428)

[1.3.2. Musculoskeletal complaints data 10](#_Toc198372429)

[1.3.3 Comorbidities 14](#_Toc198372430)

[1.3.4 Physical activity 15](#_Toc198372431)

[S2 Impact and outcomes 16](#_Toc198372432)

[2.1 Time, dose, service, demographics interaction with outcomes 16](#_Toc198372433)

[2.1.1 Minimum Clinically Important Difference (MCID) in pain and function 16](#_Toc198372434)

[2.2.2 Global Improvement 17](#_Toc198372435)

[2.2 Quality of life and personal wellbeing 18](#_Toc198372436)

[2.2.1 EuroQoL (EQ-5D) 18](#_Toc198372437)

[2.2.2 Personal wellbeing (ONS-4) 19](#_Toc198372438)

[2.3 Service acceptability and user satisfaction 22](#_Toc198372439)

[2.3.1 Stickiness 22](#_Toc198372440)

[2.3.2 Satisfaction 23](#_Toc198372441)

[2.3.3 Perceived Benefit 24](#_Toc198372442)

## Table of Figures

[Figure 1 Evaluation approach for the clinical audit 1](#_Toc197072190)

[Figure 2 How participants accessed the community service 2](#_Toc197072191)

[Figure 3 Heat map of uptake of the intervention across the United Kingdom 4](#_Toc197072192)

[Figure 4 Age distribution of participants 6](#_Toc197072193)

[Figure 5 Gender distribution of participants 7](#_Toc197072194)

[Figure 6 Gender distribution of participants 8](#_Toc197072195)

[Figure 7 Distribution of participants according to socioeconomic status form their postcode 9](#_Toc197072196)

[Figure 8 Distribution of participants according to socioeconomic status form their postcode 10](#_Toc197072197)

[Figure 9 Distribution of the all-body part reported by participants 10](#_Toc197072198)

[Figure 10 Number of participants with a comorbidity and distribution of types of conditions 14](#_Toc197072199)

[Figure 11 Baseline levels of physical activity categories form the iPAQ 15](#_Toc197072200)

[Figure 12 Global change reported by participants for the primary complaint 17](#_Toc197072201)

[Figure 13 EQ5D scores over time 18](#_Toc197072202)

[Figure 14 Change in EQ-5D scores over time 18](#_Toc197072203)

[Figure 15 ONS-4 Life satisfaction of participants over time 19](#_Toc197072204)

[Figure 16 ONS-4 Life worthwhile over time 19](#_Toc197072205)

[Figure 17 ONS-4 Happiness over time 20](#_Toc197072206)

[Figure 18 ONS-4 Anxiety over time 20](#_Toc197072207)

[Figure 19 Long term adherence >90 days 22](#_Toc197072208)

[Figure 20 Change in participant satisfaction over time 23](#_Toc197072209)

[Figure 21 The perceived benefit participant felt for their primary condition over time 24](#_Toc197072210)

## Table of Tables

[Table 1 Raw data for numbers of new registrations and application through which participants registered with the intervention 1](#_Toc197071234)

[Table 2 Session delivered per delivery of the intervention per 3-month quarters 1](#_Toc197071235)

[Table 3 The total number of sessions completed per participant 2](#_Toc197071236)

[Table 4 How participants accessed the community service 3](#_Toc197071237)

[Table 5 Uptake of the intervention by community venues 3](#_Toc197071238)

[Table 6 Number of trained facilitators for the intervention 4](#_Toc197071239)

[Table 7 Distances travelled by participants to the community venues 5](#_Toc197071240)

[Table 8 The number of uploads and impressions for the private intervention 5](#_Toc197071241)

[Table 9 Age distribution of participants 6](#_Toc197071242)

[Table 10 Gender distribution of participants 7](#_Toc197071243)

[Table 11 Distribution of ethnicity of participants 8](#_Toc197071244)

[Table 12 Distribution of participants according to socioeconomic status 9](#_Toc197071245)

[Table 13 Table showing the MSK diagnosis for the primary complaint 11](#_Toc197071246)

[Table 14 Table showing frequency of type of orthopedic surgeries in the participants 12](#_Toc197071247)

[Table 15 Table showing the distribution of the ranges of max and average pain (VAS 0-100) 13](#_Toc197071248)

[Table 16 Table showing range of difficulty with functioning (PSC 0-100) at baseline 13](#_Toc197071249)

[Table 17 Number of participants with a comorbidity and distribution of types of conditions 14](#_Toc197071250)

[Table 18 Cumulative proportion of participants reaching minimal important clinical difference in pain and functioning 17](#_Toc197071251)

[Table 19 Global change reported by participants for the primary complaint 18](#_Toc197071252)

[Table 20 Results of EQ-5D over time 19](#_Toc197071253)

[Table 21 Long-term adherence >90 days 23](#_Toc197071254)

[Table 22 Change in participant satisfaction over time 24](#_Toc197071255)

[Table 23 Raw values for perceived benefit felt by the participants 0 = no benefit 25](#_Toc197071256)

# S1 Implementation Evaluation

## 1.1 What has been delivered?

### 1.1.1 Participant registration per environment

This figure shows the number of signups completed over time, broken down into 8-month periods, and the product on which they completed their first session. There has been a large increase in signups throughout the study, particularly through the Community app.

Table 1 Raw data for numbers of new registrations and application through which participants registered with the intervention

| **Period Start** | **Period End** | **Community** | **Aqua Home** | **Land Home** |
| --- | --- | --- | --- | --- |
| 2021-05-01 | 2021-08-31 | 72 | 25 | 2 |
| 2021-09-01 | 2021-12-31 | 169 | 0 | 1 |
| 2022-01-01 | 2022-04-30 | 251 | 26 | 31 |
| 2022-05-01 | 2022-08-31 | 259 | 121 | 3 |
| 2022-09-01 | 2022-12-31 | 369 | 35 | 3 |
| 2023-01-01 | 2023-04-30 | 609 | 25 | 20 |
| 2023-05-01 | 2023-08-31 | 1187 | 39 | 18 |
| 2023-09-01 | 2023-12-31 | 1128 | 11 | 24 |

### 1.1.2 Sessions per environment

This figure shows the number of sessions completed across each of the services. We see a large increase over time in the number of Community Sessions.

Table 2 Session delivered per delivery of the intervention per 3-month quarters

| **Period Start** | **Period End** | **Community** | **Aqua Home** | **Land Home** |
| --- | --- | --- | --- | --- |
| 2021-05-01 | 2021-08-31 | 516 | 33 | 7 |
| 2021-09-01 | 2021-12-31 | 1392 | 0 | 0 |
| 2022-01-01 | 2022-04-30 | 2232 | 75 | 128 |
| 2022-05-01 | 2022-08-31 | 2914 | 648 | 164 |
| 2022-09-01 | 2022-12-31 | 3446 | 243 | 134 |
| 2023-01-01 | 2023-04-30 | 5303 | 133 | 229 |
| 2023-05-01 | 2023-08-31 | 9512 | 216 | 78 |
| 2023-09-01 | 2023-12-31 | 13229 | 101 | 262 |

### 1.1.3 Adherence

4,429 participants have completed 40,995 sessions at an average of 9.26 sessions per participant. 3,231 (73%) participants have completed more than 1 session and with an average of 102.1 days between their first and last session.

Table 3 The total number of sessions completed per participant

| **Sessions** | **participants** | **% of participants** |
| --- | --- | --- |
| Single Session | 1198 | 27.05% |
| 2-5 Sessions | 1551 | 35.02% |
| 6-10 Sessions | 730 | 16.48% |
| 11-20 Sessions | 478 | 10.79% |
| 21+ Sessions | 472 | 10.66% |

## 1.2 How has it been delivered?

### 1.2.1 How did you hear about the service?

When participants sign up to the service through the community service, they are asked how they heard about the service. The majority (63.7%) come from Leisure Venues or through GP/Physio referrals. The private services did not collect this data but those has started since Quarter 2 in 2024.


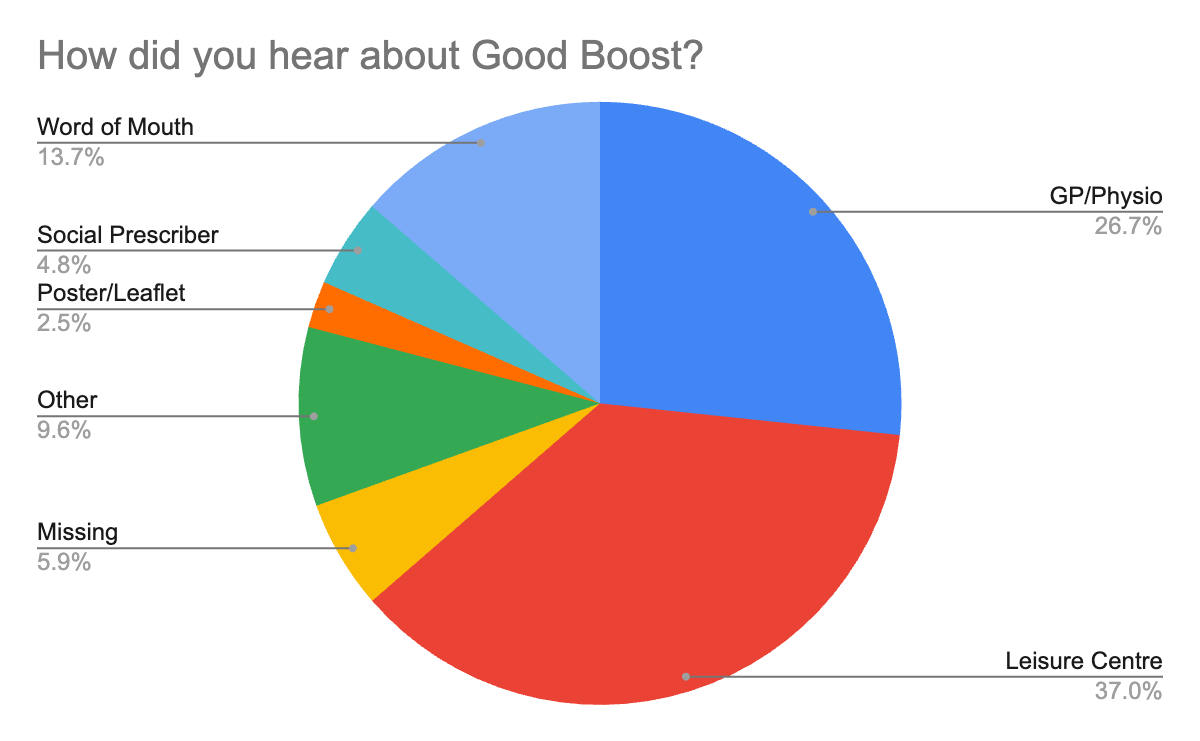


Figure 2 How participants accessed the community service

Table 4 How participants accessed the community service

| **How Heard** | **Signups** | **%** |
| --- | --- | --- |
| GP/Physio | 1043 | 26.68% |
| Leisure venue | 1445 | 36.96% |
| Missing | 229 | 5.86% |
| Other | 375 | 9.59% |
| Poster/Leaflet | 98 | 2.51% |
| Social Prescriber | 186 | 4.76% |
| Word of Mouth | 534 | 13.66% |
| Not asked | 519 | N/A |

### 1.2.2 Venue recruitment (Community)

This figure shows the cumulative count of Venues that have run sessions. By 2023-12-31 there were 136 Venues that had run leisure Venues. Of those 136 venues 8 ceased delivering sessions.

Table 5 Uptake of the intervention by community venues

| **Period Start Date** | **Period End Date** | **New Venues** | **Cumulative Count of Venues** |
| --- | --- | --- | --- |
| 2021-05-01 | 2021-08-31 | 9 | 9 |
| 2021-09-01 | 2021-12-31 | 14 | 23 |
| 2022-01-01 | 2022-04-30 | 7 | 30 |
| 2022-05-01 | 2022-08-31 | 2 | 32 |
| 2022-09-01 | 2022-12-31 | 17 | 49 |
| 2023-01-01 | 2023-04-30 | 17 | 66 |
| 2023-05-01 | 2023-08-31 | 37 | 103 |
| 2023-09-01 | 2023-12-31 | 33 | 136 |

### 1.2.3 Number of facilitators trained

Count of facilitators who have completed training over time.

Table 6 Number of trained facilitators for the intervention

| **Period Start Date** | **Period End Date** | **New Facilitators** |
| --- | --- | --- |
| 2021-05-01 | 2021-08-31 | 13 |
| 2021-09-01 | 2021-12-31 | 6 |
| 2022-01-01 | 2022-04-30 | 8 |
| 2022-05-01 | 2022-08-31 | 13 |
| 2022-09-01 | 2022-12-31 | 56 |
| 2023-01-01 | 2023-04-30 | 170 |
| 2023-05-01 | 2023-08-31 | 80 |
| 2023-09-01 | 2023-12-31 | 134 |

### 1.2.4 Location hot spots for uptake

This map shows the distribution of leisure Venues running sessions around the United Kingdom. The larger the circle, the more sessions have been completed at a given venue.


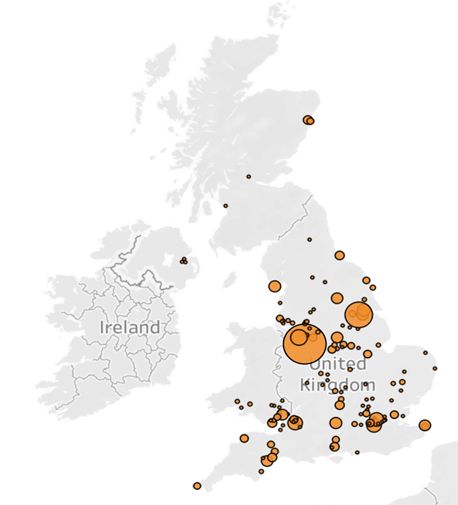


Figure 3 Heat map of uptake of the intervention across the United Kingdom

### 1.2.5 Location - Distance to Registration Venue (Community)

The following plot shows the distance from the participants’ home postcode to the postcode of the venue where they completed their first community session (as the crow flies). We have matched 84% of participants. participants with a distance of 100km +(15 participants) have not been included as it is presumed they have either moved or inputted the postcode incorrectly.

Table 7 Distances travelled by participants to the community venues

| Size | Participants | % |
| --- | --- | --- |
| 0-1km | 384 | 10.33% |
| 1.01-2km | 669 | 17.99% |
| 2.01-3km | 558 | 15.00% |
| 3.01-4km | 438 | 11.78% |
| 4.01-5km | 324 | 8.71% |
| 5.01-10km | 790 | 21.24% |
| 10.01-20km | 425 | 11.43% |
| 20km+ | 131 | 3.52% |

### 1.2.6 Private (Home) intervention uptake

The 2 home apps are available on the App Store and Google Play Store. However, impression and download figures for the App Store are displayed below:

Table 8 The number of uploads and impressions for the private intervention

|  | Impressions | Downloads |
| --- | --- | --- |
| Land | 37600 | 1900 |
| Aqua | 5100 | 835 |

## 1.3 To Whom has it been delivered?

### 1.3.1 Demographics

#### 1.3.1.1 Age

participants broken down by age group. NB participants aged over 100 have been removed as the age has likely been inputted erroneously.


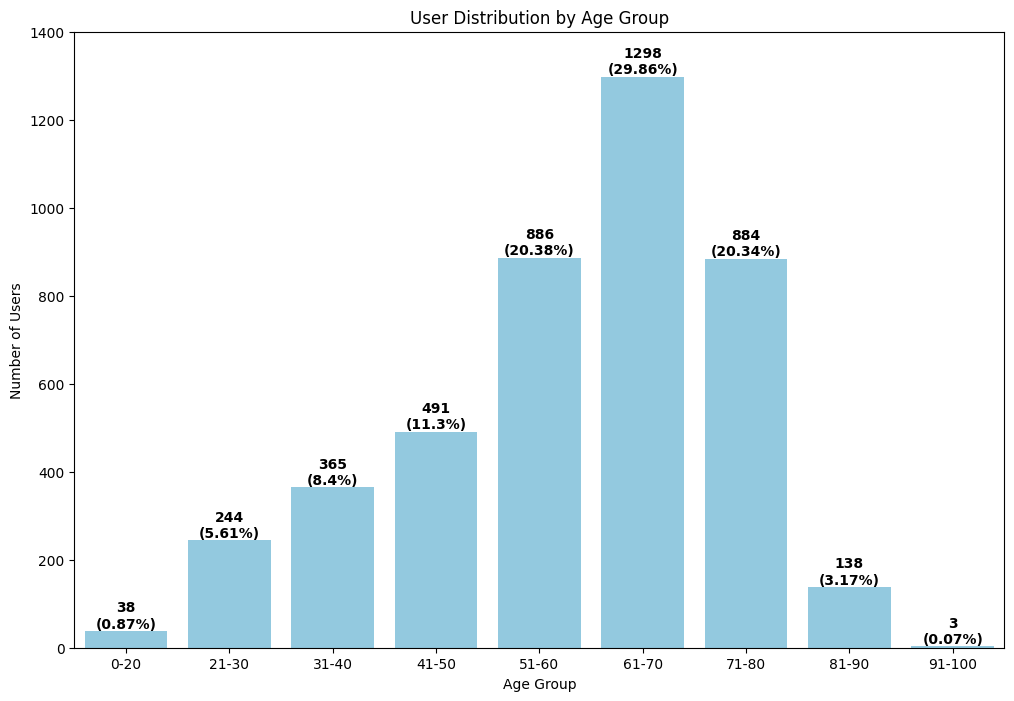


Figure 4 Age distribution of participants

Table 9 Age distribution of participants

| Age Group | participants | % |
| --- | --- | --- |
| 0-20 | 38 | 0.87% |
| 21-30 | 244 | 5.61% |
| 31-40 | 365 | 8.40% |
| 41-50 | 491 | 11.30% |
| 51-60 | 886 | 20.38% |
| 61-70 | 1,298 | 29.86% |
| 71-80 | 884 | 20.34% |
| 81-90 | 138 | 3.17% |
| 91-100 | 3 | 0.07% |

#### 1.3.1.2 Gender


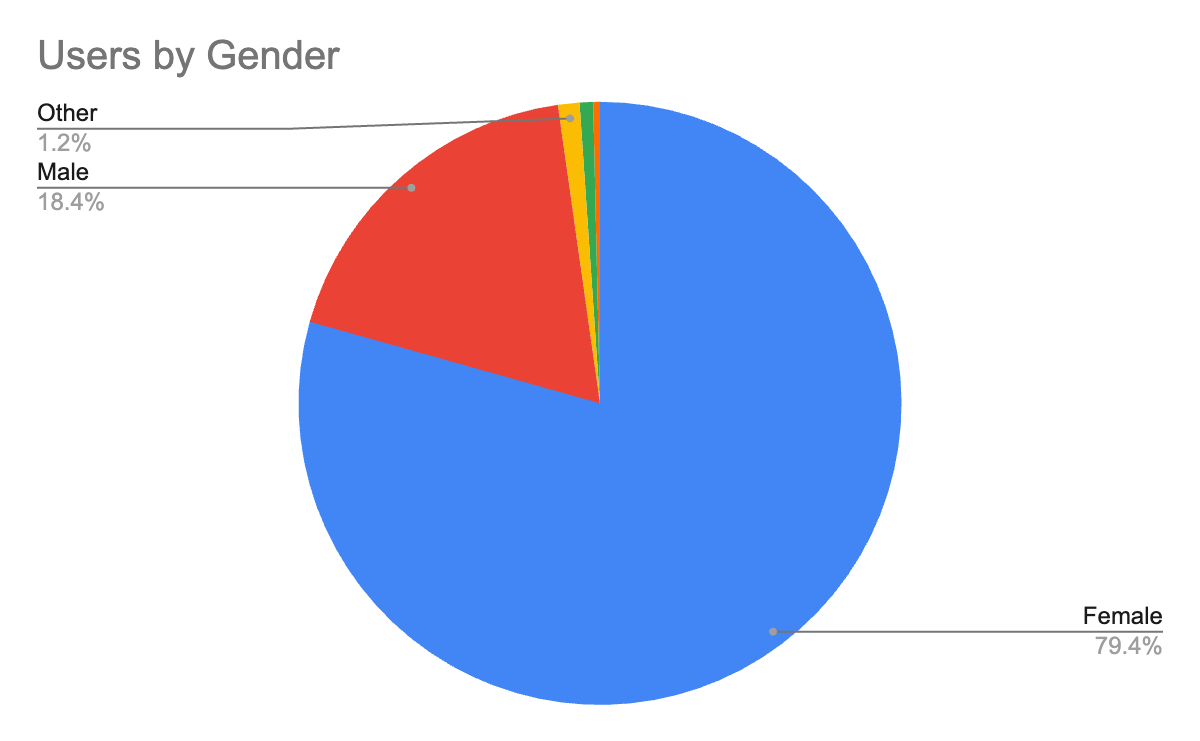


Figure 5 Gender distribution of participants

Table 10 Gender distribution of participants

| **Gender** | **participants** | **% of participants** |
| --- | --- | --- |
| Female | 3515 | 79.36% |
| Male | 815 | 18.40% |
| Other | 51 | 1.15% |
| Prefer not to say | 32 | 0.72% |
| Unknown | 16 | 0.36% |

#### 1.3.1.3 Ethnicity

participants are asked their ethnicity upon signing up. The majority identify as white but a significant minority have different ethnicities.


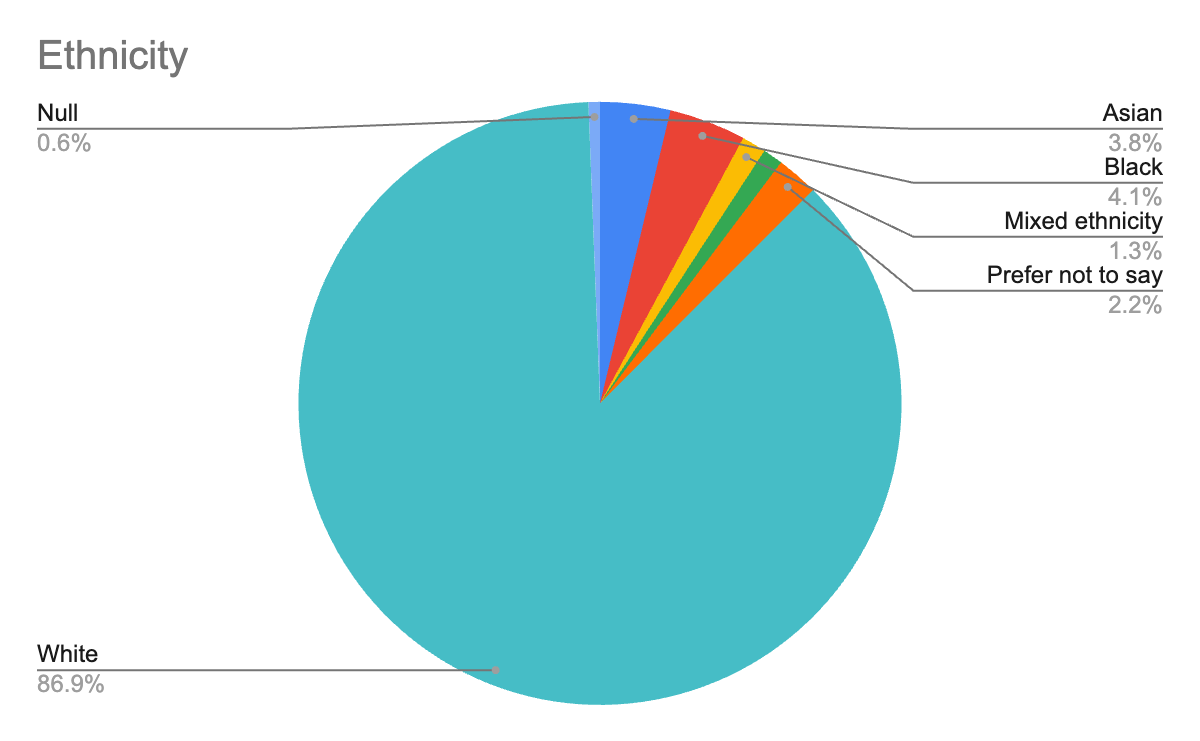


Figure 6 Gender distribution of participants

Table 11 Distribution of ethnicity of participants

| **Ethnicity** | **Participants** | **%** |
| --- | --- | --- |
| **Asian** | 147 | 3.76% |
| **Black** | 161 | 4.12% |
| **Mixed ethnicity** | 51 | 1.30% |
| **Other ethnic group** | 42 | 1.07% |
| **Prefer not to say** | 87 | 2.23% |
| **White** | 3398 | 86.91% |
| **Null** | 24 | 0.61% |
| **Not asked** | 519 |  |

#### 1.3.1.5 Socio-Economic Status

This figure displays the socioeconomic status of participants. These values are approximated by using participants’ home postcodes and approximating the Index of Multiple Deprivation using the Consumer Data Research (2019)^^[[1]](#footnote-1)^^ venue’s socio economic data. 1 Relates to participants in the lowest income areas and 10 represents the highest.


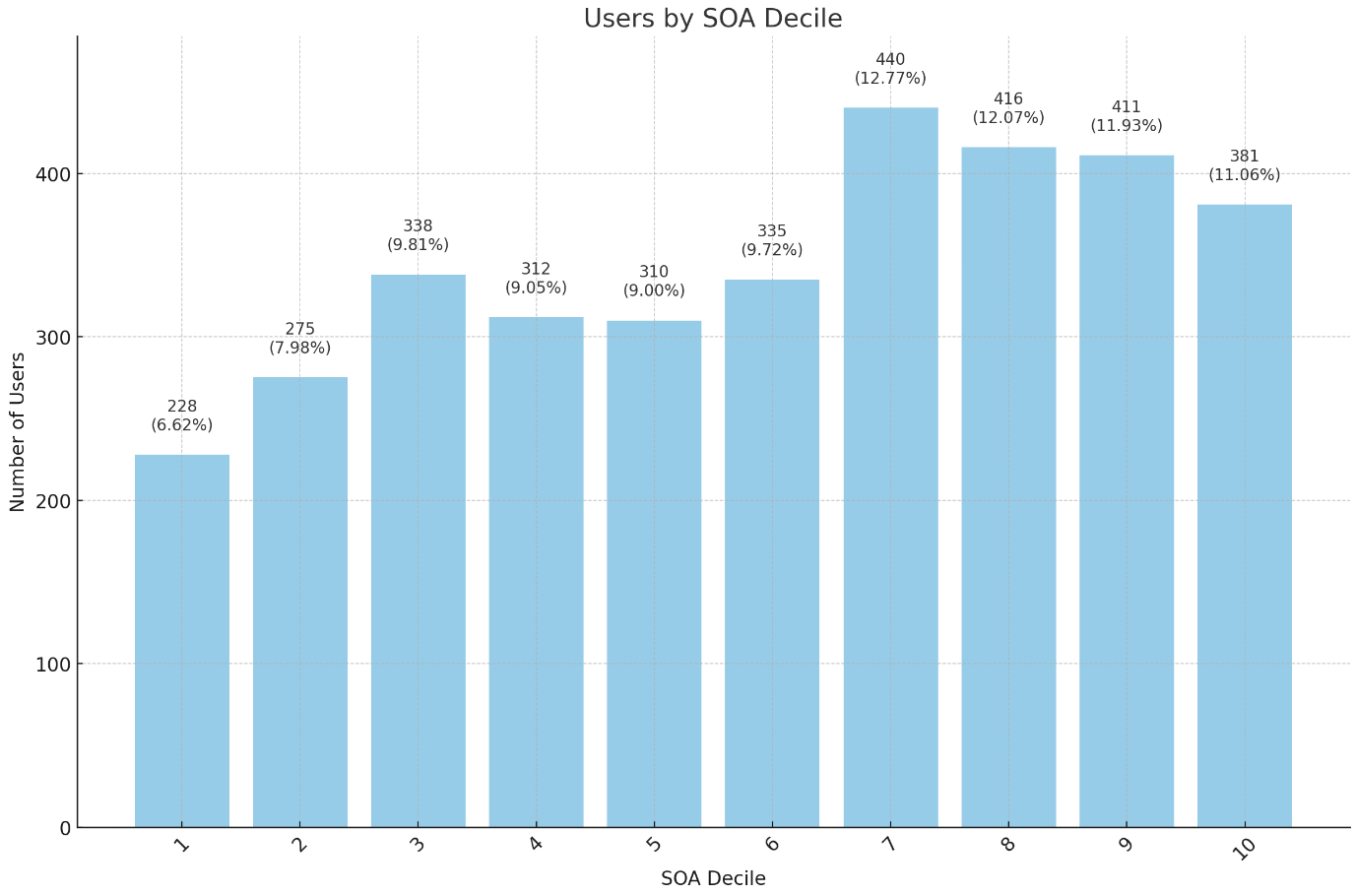


Figure 7 Distribution of participants according to socioeconomic status form their postcode

Table 12 Distribution of participants according to socioeconomic status

| **SOA_decile** | **participants** | **% of participants** |
| --- | --- | --- |
| **1** | 228 | 6.62% |
| **2** | 275 | 7.98% |
| **3** | 338 | 9.81% |
| **4** | 312 | 9.05% |
| **5** | 310 | 9.00% |
| **6** | 335 | 9.72% |
| **7** | 440 | 12.77% |
| **8** | 416 | 12.07% |
| **9** | 411 | 11.93% |
| **10** | 381 | 11.06% |

### 1.3.2. Musculoskeletal complaints data

#### 1.3.2.1 Body parts affected by MSK conditions

3,608 participants have registered 5530 complaints at an average of 1.53 complaints per participant. Primary complaints are the first complaints that a participant register into the system. The most common primary by part affected by MSK conditions the knee with 33% of participants.


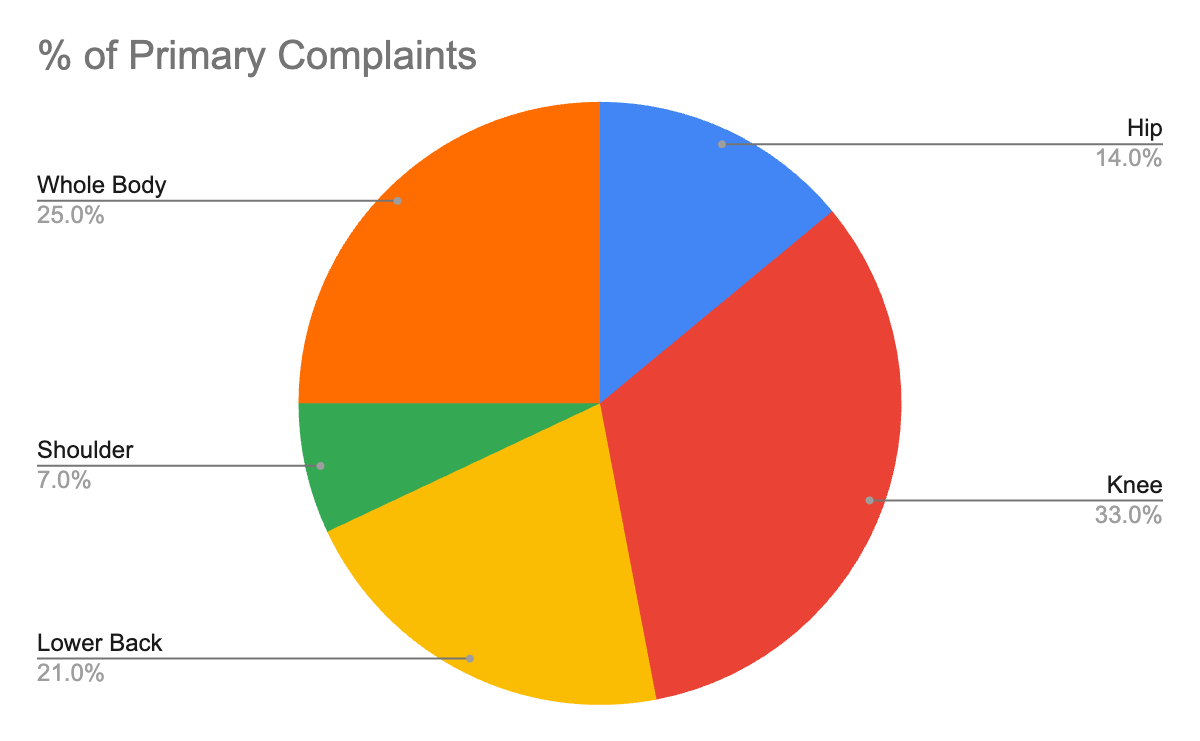


Figure 8 Distribution of participants according to socioeconomic status form their postcode


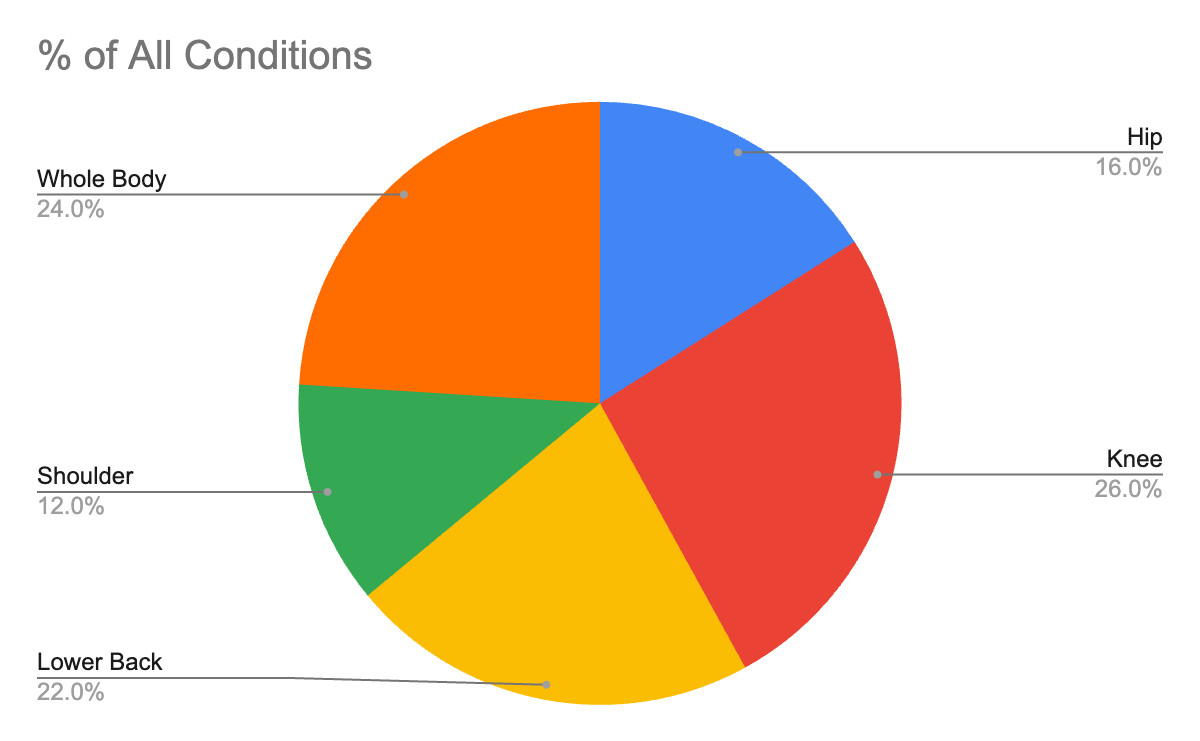


Figure 9 Distribution of the all-body part reported by participants

#### 1.3.2.2 MSK diagnosis

Table 13 Table showing the MSK diagnosis for the primary complaint

| **Diagnosis** | **Count** | **Percentage** |
| --- | --- | --- |
| Axial Spondyloarthritis | 44 | 1.4 |
| Bruise | 1 | 0.0 |
| Bursitis | 23 | 0.7 |
| Chronic Fatigue Syndrome | 31 | 1.0 |
| Chronic Pain Syndrome | 28 | 0.9 |
| Disc Injury | 72 | 2.2 |
| Diffuse Idiopathic Skeletal Hyperostosis | 1 | 0.0 |
| Fibromyalgia | 178 | 5.5 |
| Fracture (stress) | 10 | 0.3 |
| Fracture (trauma) | 23 | 0.7 |
| Frozen Shoulder | 31 | 1.0 |
| Hip Dislocation and non-surgical relocation | 1 | 0.0 |
| Inflammatory Arthritis | 173 | 5.4 |
| Instability | 5 | 0.2 |
| Ligament Injury | 14 | 0.4 |
| Meniscus Injury | 27 | 0.8 |
| Muscle injury | 7 | 0.2 |
| Myofascial Syndrome | 2 | 0.1 |
| No specific diagnosis | 741 | 23.1 |
| Osgood-Schlatter's Disease | 1 | 0.0 |
| Osteoarthritis | 942 | 29.4 |
| Osteopenia | 24 | 0.7 |
| Osteoporosis | 86 | 2.7 |
| Other (Specified) | 391 | 12.2 |
| Patella Subluxation/Dislocation | 3 | 0.1 |
| Patellofemoral Pain/Chondromalacia Patella | 7 | 0.2 |
| Polymyalgia Rheumatica | 13 | 0.4 |
| Psoriatic Arthritis | 26 | 0.8 |
| Reactive Arthritis | 24 | 0.7 |
| Rheumatoid Arthritis | 117 | 3.6 |
| Rotator Cuff Tendinopathy | 13 | 0.4 |
| Scheuermann's Disease | 2 | 0.1 |
| Sciatica | 57 | 1.8 |
| Shoulder dislocation | 3 | 0.1 |
| Shoulder impingement syndrome | 9 | 0.3 |
| Spondylosis | 12 | 0.4 |
| Stenosis | 38 | 1.2 |
| Tendinopathy | 28 | 0.9 |
| **Total** | **3208** |  |

1.3.2.3 Orthopedic surgery

Table 14 Table showing frequency of type of orthopedic surgeries in the participants

| **Surgery** | **Frequency (n)** |
| --- | --- |
| Arthroscopy/Debridement | 29 |
| Discectomy | 8 |
| Foraminotomy/Facetectomy | 1 |
| Fracture internal fixation | 11 |
| Meniscus Partial/Full Removal | 5 |
| Osteotomy | 3 |
| Partial Hip Replacement | 3 |
| Partial Knee Replacement | 19 |
| Rotator cuff repair | 5 |
| Spinal fusion (lumbar spine) | 19 |
| Spinal laminectomy/spinal decompression | 16 |
| Subacromial decompression | 2 |
| Total Hip Replacement | 68 |
| Total Hip Replacement (Revision) | 6 |
| Total Knee Replacement | 125 |
| Total Knee Replacement (Revision) | 20 |
| Other (Specified) | 60 |
| **Total** | **400** |

#### 1.3.2.5 Symptoms (Pain)

Upon registering a complaint, participants are asked what the average and max pain level has been for the particular body part in the last 24 hours on a scale from 0-100. The results at baseline are displayed here in a box plot.

Table 15 Table showing the distribution of the ranges of max and average pain (VAS 0-100)

|  |  | **Max Pain** | | | | | **Average pain** | | | | |
| --- | --- | --- | --- | --- | --- | --- | --- | --- | --- | --- | --- |
| **Body Part** | **n** | **Max** | **Min** | **Median** | **IQR- 25** | **IQR - 75** | **Max** | **Min** | **Median** | **IQR-25** | **IQR-75** |
| Combined | 3608 | 100 | 0 | 48 | 29 | 64 | 100 | 0 | 44 | 26 | 60 |
| Hip | 498 | 100 | 0 | 47 | 30 | 63 | 100 | 0 | 46 | 26.25 | 60 |
| Knee | 1196 | 100 | 0 | 47.5 | 28 | 62 | 100 | 0 | 42 | 26 | 57 |
| Lower back | 766 | 100 | 0 | 49 | 30 | 64 | 100 | 0 | 46 | 29 | 62 |
| Shoulder | 261 | 100 | 0 | 43 | 21 | 59 | 100 | 0 | 37 | 20 | 55 |
| Generalised | 887 | 100 | 0 | 50 | 29 | 67 | 100 | 0 | 47 | 26 | 63 |

#### 1.3.2.6 Symptoms Function

Upon registering a body part they want to focus the exercise on participants are asked about a specific activity in their life that they find difficult and to rate that difficulty from 0 - 100. The results are displayed below:

Table 16 Table showing range of difficulty with functioning (PSC 0-100) at baseline

| **Body Part** | **n** | **Max** | **Min** | **Median** | **IQR-25** | **IQR-75** |
| --- | --- | --- | --- | --- | --- | --- |
| Combined | 3607 | 100 | 0 | 67 | 46 | 83 |
| Hip | 498 | 100 | 0 | 67 | 46 | 83 |
| Knee | 1196 | 100 | 0 | 68 | 48 | 83 |
| Lower back | 766 | 100 | 0 | 66 | 45.25 | 80 |
| Shoulder | 260 | 100 | 0 | 62.5 | 39 | 78.25 |
| Generalised | 887 | 100 | 0 | 67 | 47 | 85 |

### 1.3.3 Comorbidities

Participants are asked a variety of questions about their general health. Responses to these questions are recorded in this figure. 76% of participants report at least one comorbidity.


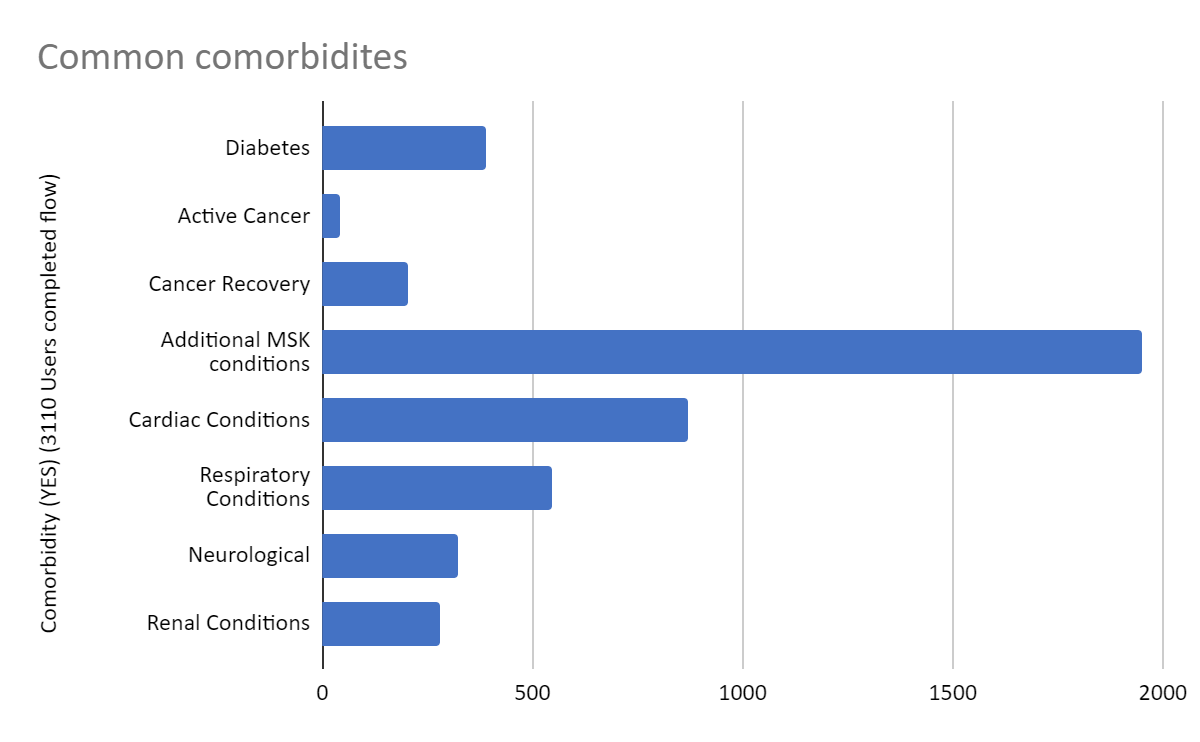


Figure 10 Number of participants with a comorbidity and distribution of types of conditions

Table 17 Number of participants with a comorbidity and distribution of types of conditions

| **Comorbidity (n=3110)** | **n** | **%** |
| --- | --- | --- |
| Any | 2443 | 78.6 |
| Diabetes | 388 | 12.5 |
| Cancer (Active) | 43 | 1.4 |
| Cancer Recovery | 205 | 6.6 |
| Additional MSK conditions | 1949 | 62.7 |
| Cardiac Conditions | 872 | 28.0 |
| Respiratory Conditions | 545 | 17.5 |
| Neurological | 323 | 10.4 |
| Renal Conditions | 279 | 9.0 |

### 1.3.4 Physical activity

The IPAQ survey ascertains somebody’s physical activity levels by asking questions about their physical activity in the previous week.The survey is asked at baseline. The responses are collated into a physical activity level of Low, Medium or High and the results are displayed below:


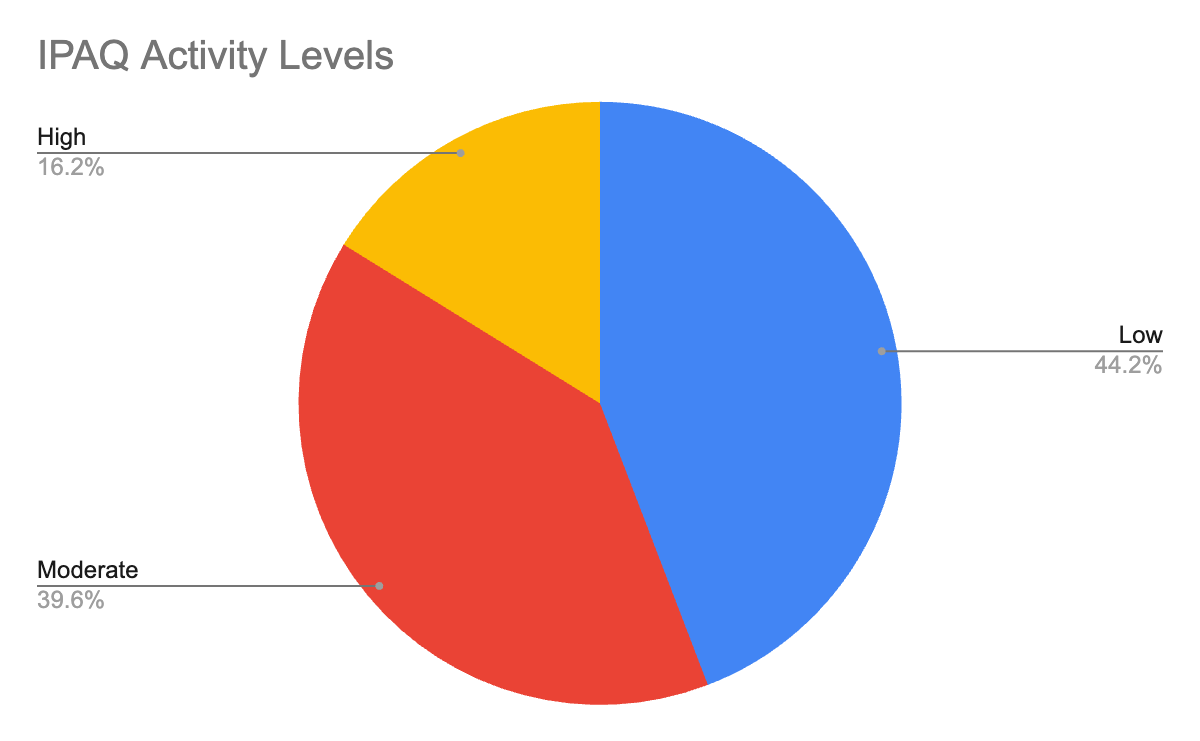


Figure 11 Baseline levels of physical activity categories form the iPAQ

# S2 Impact and outcomes

*All data refers to UK based participants who signed up between 1^st^ May 2021 and 31^st^ December 2023 and completed at least one exercise session within those dates.*

## 2.1 Time, dose, service, demographics interaction with outcomes

### 2.1.1 Minimum Clinically Important Difference (MCID) in pain and function

Study X states that a 15% reduction in a self-reported pain metric corresponds to a minimal clinical important difference (MCID) in pain. With long term health conditions pain levels are likely to fluctuate. We track the % of users who have experienced a MCID in their Max Pain score, Average Pain score and PSC Score at or before a given date. The results are plotted below. In total 55.1% of users who have reported outcome values for Average Pain have experienced an MCID, 45.9% have experienced an MCID in average pain and 48.4 % have experienced an MCID in max pain. This is for primary complaint.

Table 18 Cumulative proportion of participants reaching minimal important clinical difference in pain and functioning

| **Time points from Baseline** | **Max Pain** | | **Average pain** | | **Function (PSC)** | | **Any effect** | |
| --- | --- | --- | --- | --- | --- | --- | --- | --- |
|  | **n** | **%** | **n** | **%** | **n** | **%** | **n** | **%** |
| 20 days | 13 | 0.8 | 15 | 0.9 | 0 | 0.0 | 20 | 1.2 |
| 40 days | 341 | 20.4 | 322 | 19.3 | 424 | 25.4 | 601 | 36.0 |
| 60 days | 556 | 33.3 | 527 | 31.6 | 668 | 40.0 | 943 | 56.5 |
| 80 days | 635 | 38.1 | 605 | 36.2 | 759 | 45.5 | 1043 | 62.5 |
| 100 days | 713 | 42.7 | 672 | 40.2 | 834 | 50.0 | 1132 | 67.8 |
| 120 days | 734 | 44.0 | 691 | 41.4 | 852 | 51.1 | 1151 | 68.9 |
| 140 days | 742 | 44.5 | 705 | 42.2 | 868 | 52.0 | 1169 | 70.0 |
| 160 days | 759 | 45.5 | 722 | 43.2 | 886 | 53.1 | 1193 | 71.4 |
| 180 days | 768 | 46.0 | 731 | 43.8 | 894 | 53.6 | 1200 | 71.9 |
| 180+ days | 807 | 48.4 | 767 | 45.9 | 920 | 55.1 | 1233 | 73.8 |

### 2.2.2 Global Improvement


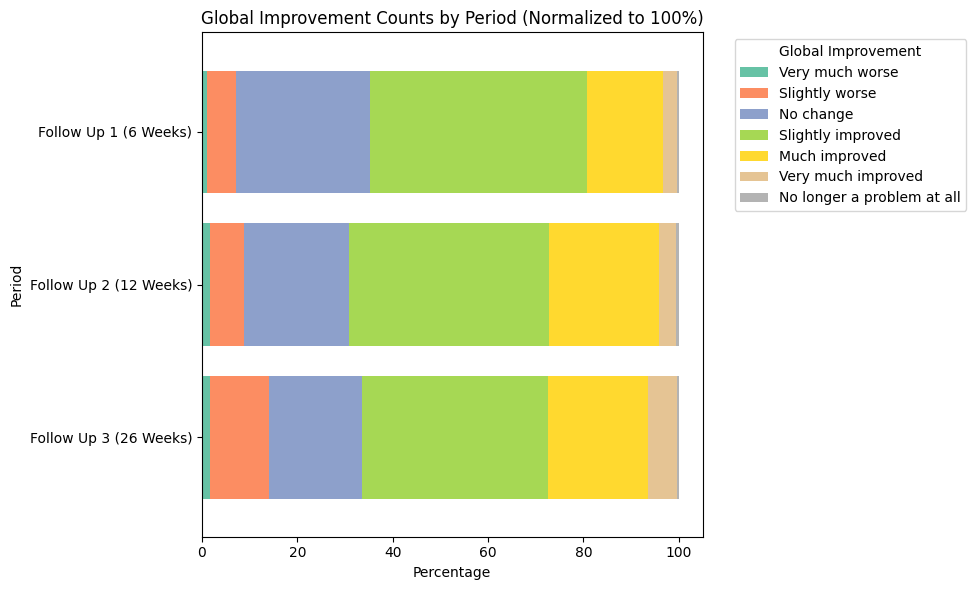


Figure 12 Global change reported by participants for the primary complaint

Table 19 Global change reported by participants for the primary complaint

| **Follow-up point** | **Very Much Worse** | | **Slightly Worse** | | **No Change** | | **Slightly Improved** | | **Much Improved** | | **Very Much Improved** | | **No Longer a Problem at All** | |
| --- | --- | --- | --- | --- | --- | --- | --- | --- | --- | --- | --- | --- | --- | --- |
|  | n | % | n | % | n | % | n | % | n | % | n | % | n | % |
| 6 Weeks | 14 | 1.0 | 84 | 6.07 | 389 | 28.1 | 629 | 45.5 | 220 | 15.9 | 43 | 3.1 | 4 | 0.3 |
| 12 Weeks | 13 | 1.7 | 54 | 7.17 | 165 | 21.9 | 316 | 42.0 | 174 | 23.1 | 27 | 3.6 | 4 | 0.5 |
| 26 Weeks | 4 | 1.7 | 29 | 12.5 | 45 | 19.3 | 91 | 39.1 | 49 | 21.0 | 14 | 6.0 | 1 | 0.4 |

## 2.2 Quality of life and personal wellbeing

### 2.2.1 EuroQoL (EQ-5D)

The answers to the EQ5D survey can be mapped onto a scale from 0 to 1 using (LINK). The results are plotted in figure 5 below. Due to small n in follow up questions a statistical analysis was not performed


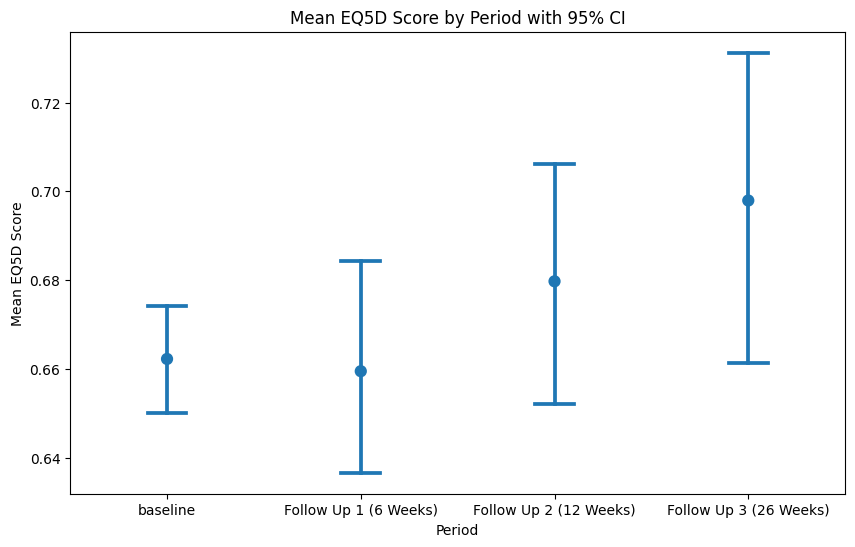

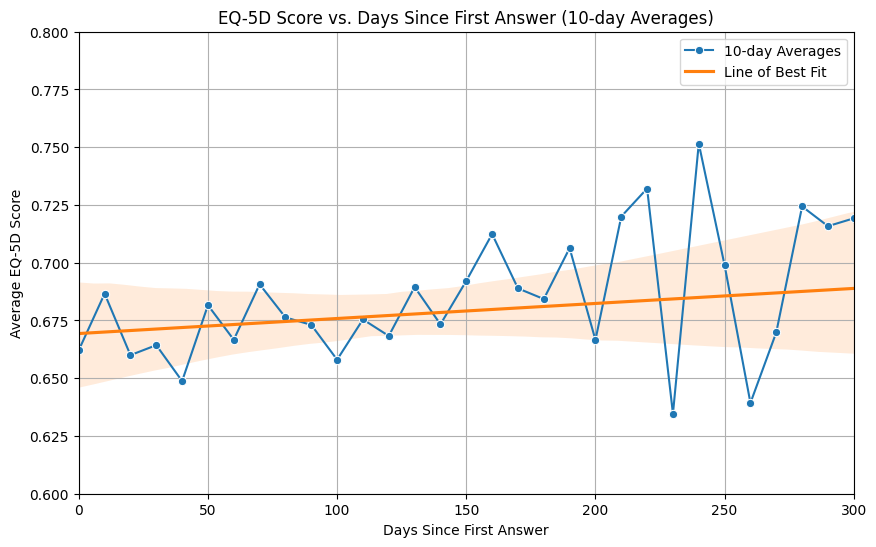


Figure 13 EQ5D scores over time

Figure 14 Change in EQ-5D scores over time

Table 20 Results of EQ-5D over time

| Period | n | Mean | 95% Confidence Interval Lower | 95% Confidence Interval Upper |
| --- | --- | --- | --- | --- |
| Baseline | 1136 | 0.662 | 0.650 | 0.674 |
| 6 Weeks | 331 | 0.660 | 0.637 | 0.682 |
| 12 Weeks | 197 | 0.680 | 0.652 | 0.707 |
| 26 Weeks | 113 | 0.698 | 0.663 | 0.733 |

### 2.2.2 Personal wellbeing (ONS-4)

Since March 2023 users have been asked to fill out the ONS4 health ad wellbeing survey. It is asked at baseline, 6 weeks, 12 weeks and 26 week intervals. The questions are:

- Overall, how satisfied are you with your life nowadays?
- Overall, to what extent do you feel the things you do in your life are worthwhile?
- Overall, how happy did you feel yesterday?
- Overall, how anxious did you feel yesterday?

#### 2.2.2.1 ONS-4 Life Satisfaction


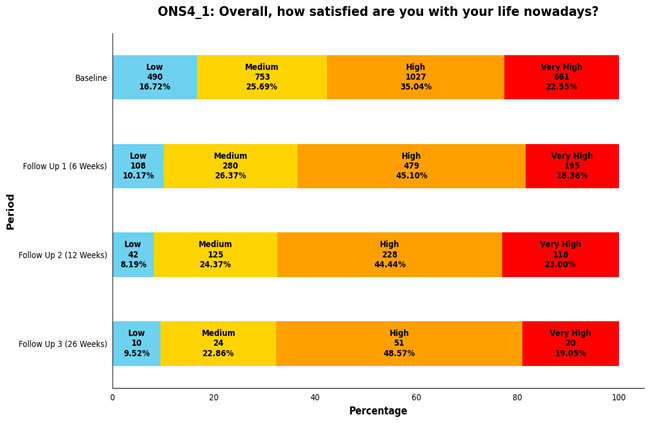


Figure 15 ONS-4 Life satisfaction of participants over time

#### 2.2.2.2 ONS-4 Life worthwhile


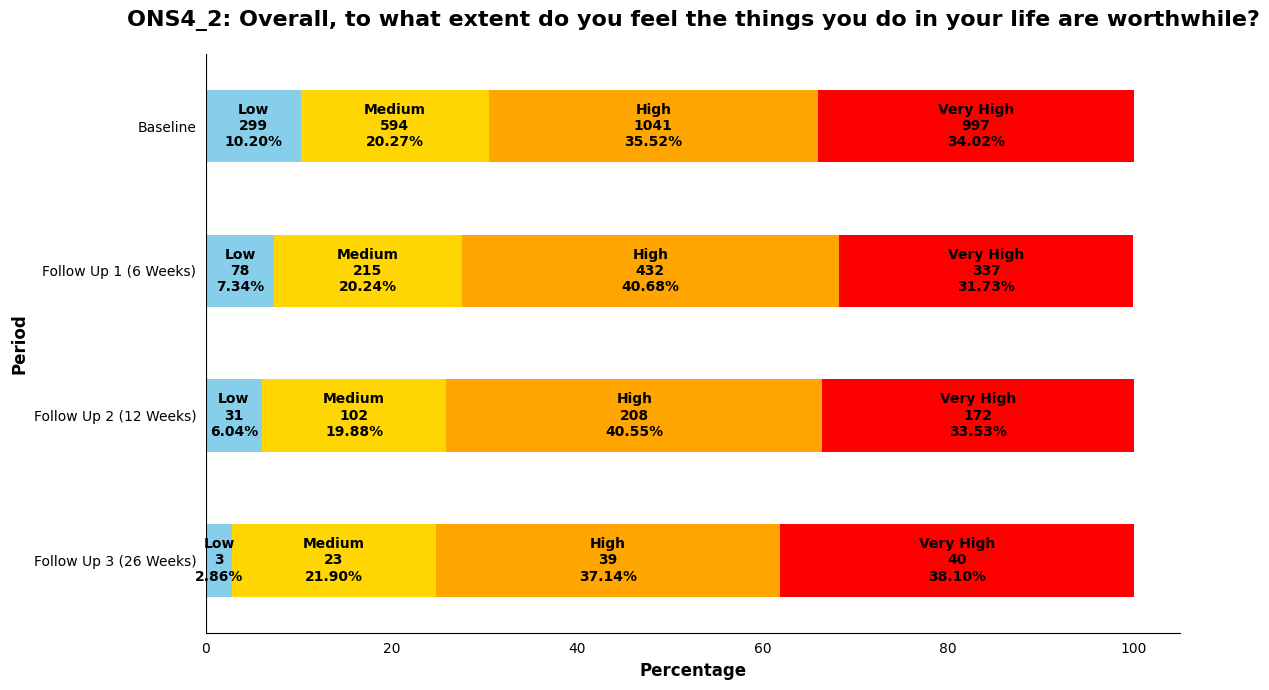


Figure 16 ONS-4 Life worthwhile over time

#### 2.2.2.3 ONS-4 Happiness


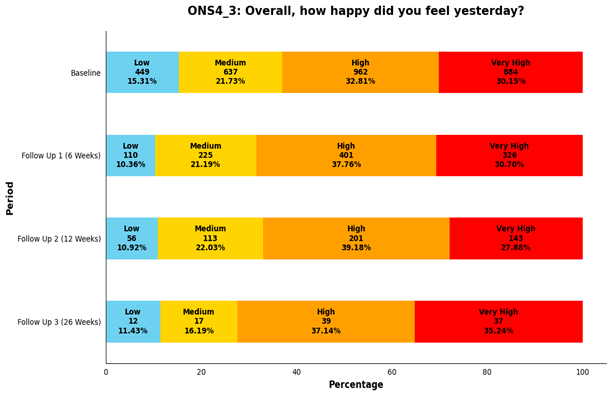


Figure 17 ONS-4 Happiness over time

#### 2.2.2.4 ONS-4 Anxiety


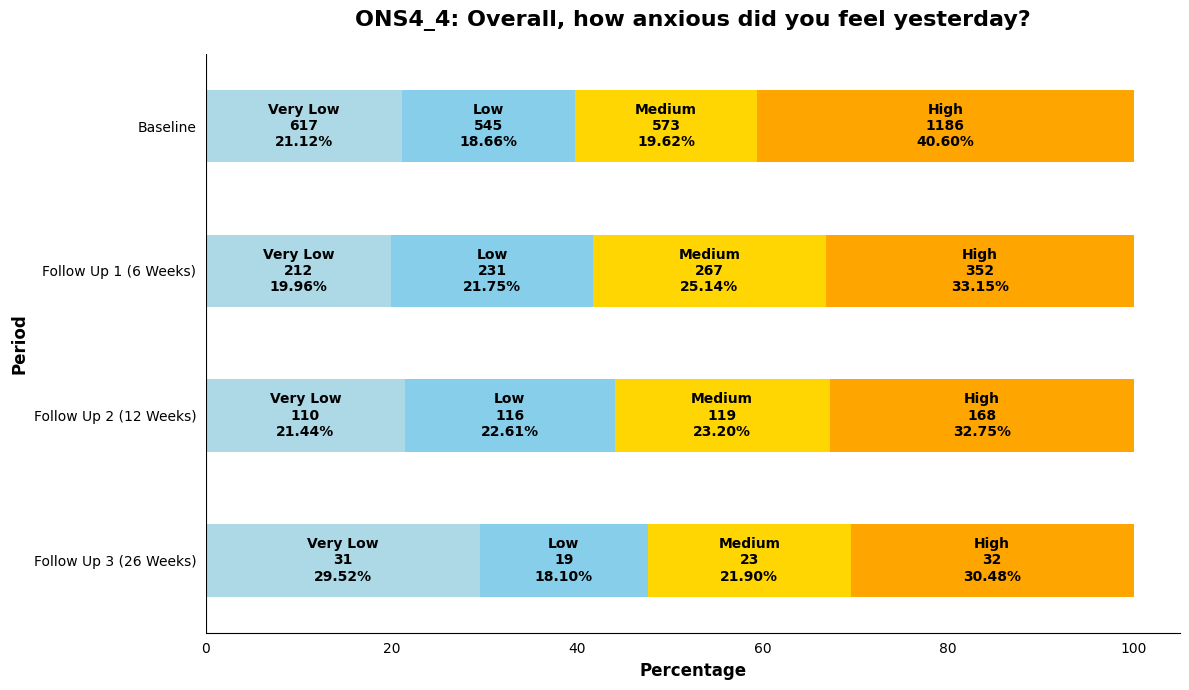


Figure 18 ONS-4 Anxiety over time

#### 2.2.5.1 ONS4 Analysis:

Based on the raw data presented in section a deeper statistic test was performed.

McNemar's test is a useful tool here as it is designed to analyse paired data where the same subjects are measured at two different time points. In this scenario, each users’ response is categorised as Low or High at both baseline and outcome, making the data inherently paired. To ensure this we have taken users first and last responses to these questions as baseline and outcome and only looked at users who have answered the survey twice or more times. This test is necessary to determine if there is a statistically significant change in the proportion of a particular response category (in this case, "High") between the two time points.

The results of McNemar's test on the responses to the first ONS4 question: Overall, how satisfied are you with your life nowadays? The proportion of users responding High/Very High increased from 57.1% at baseline to 65.7% at outcome. This gave a test statistic of 26.41 and a p-value <0.001, indicating a highly significant change in the proportion of users responding High/Very High from baseline to outcome. The low p-value suggests that the observed change is unlikely to have occurred by chance, affirming the significance of the intervention between the two time points.

The responses to the other 3 questions alluded to similar improvements. We saw a test statistic of 7.17 and a p-value of 0.007 indicating that the proportion of people responding High to Overall, to what extent do you feel the things you do in your life are worthwhile? Improved. We saw a test stat of 10.3 and a p-value of 0.001 indicating that the proportion of people responding High/Very High to “Overall, how happy did you feel yesterday?” improved. In addition to this we saw the proportion of people responding High’ to “Overall, how anxious did you feel yesterday?” decrease with a test statistic of 14.57 and p value of <0.0001.

Cumulative Link Mixed Model (CLMM) analysis shows that participants who have seen changes in their ONS4 scores from Baseline to Follow Up. This method of analysis allows us to look at the change in the ranked categorical answers which users give to the ONS4 questions ranging from ‘Very Low’ to ‘Very High’ Fitting a Cumulative Link Mixed Model with the Laplace Approximation to the results of each of the ONS4 survey showed significant improvements in life satisfaction at both 6 and 12 week follow ups (p values of 0.007 and <0.001 respectively). At the 6 week follow up we saw an increase in follow up (p-value: 0.003). Finally we saw a statistically significant decrease in Anxiety at 6 weeks, 12 weeks and 26 weeks (p-values of 0.012,0.009 and 0.011).

## 2.3 Service acceptability and user satisfaction

### 2.3.1 Stickiness

This figure tracks the number of signups over time against the % of those signups that then went on to complete 2 session 90 or more days apart.


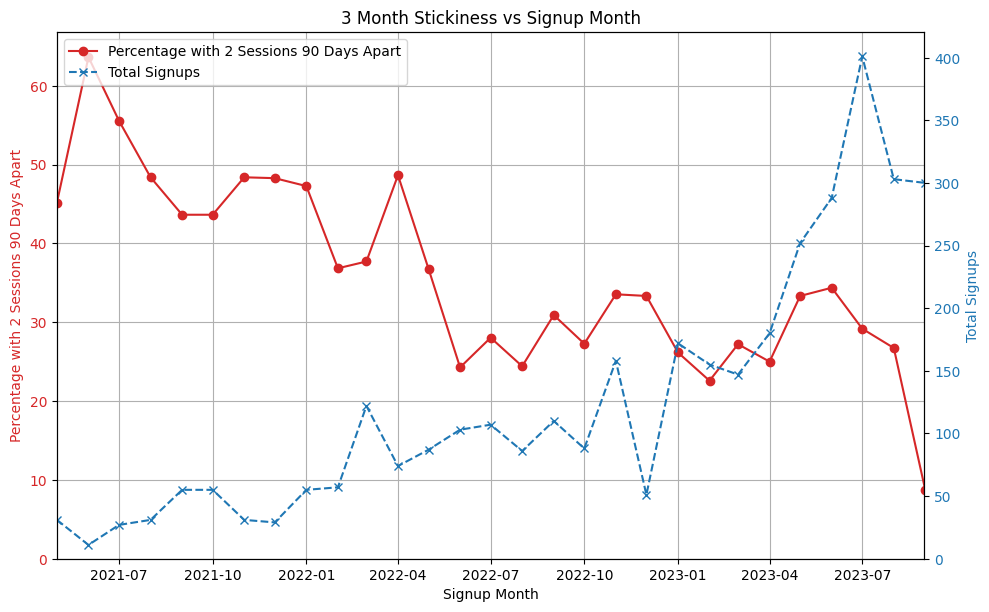


Figure 19 Long term adherence >90 days

Table 21 Long-term adherence >90 days

| **Time Period** | **Signups** | **Users With 2 Sessions 90+ Days Apart** | **% of Users with 3 Month Stickiness** |
| --- | --- | --- | --- |
| 2021-05-01 to 2021-08-31 | 100 | 51 | 51.00% |
| 2021-09-01 to 2021-12-31 | 170 | 77 | 45.29% |
| 2022-01-01 to 2022-04-30 | 308 | 129 | 41.88% |
| 2022-05-01 to 2022-08-31 | 383 | 108 | 28.20% |
| 2022-09-01 to 2022-12-31 | 407 | 128 | 31.45% |
| 2023-01-01 to 2023-04-30 | 654 | 165 | 25.23% |
| 2023-05-01 to 2023-08-31 | 1244 | 381 | 30.63% |
| 2023-09-01 to 2023-09-30 | 300 | 26 | 8.67% |

### 2.3.2 Satisfaction

After a session, users are asked to rate it from 0 to 5 stars. The results are displayed here.


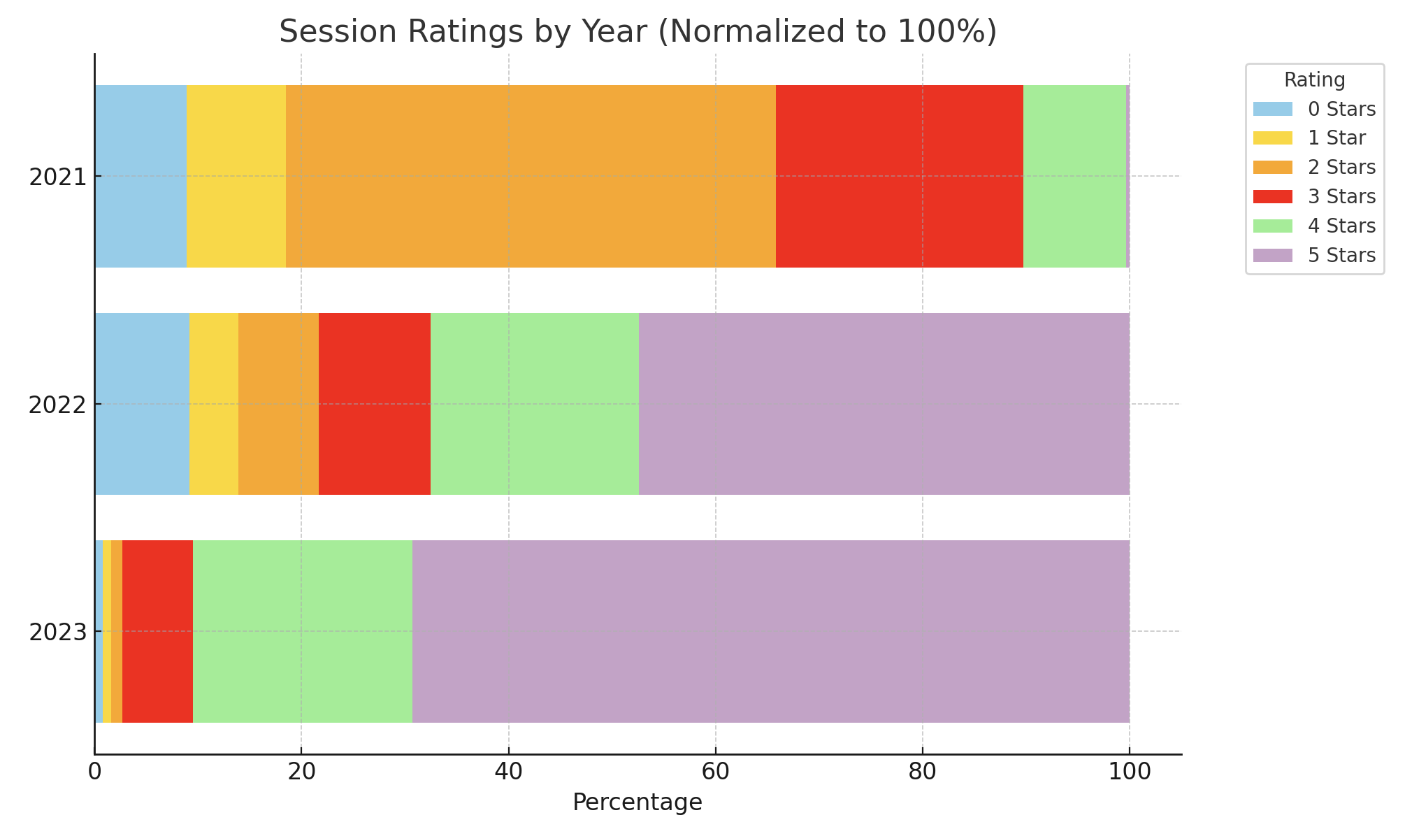


Figure 20 Change in participant satisfaction over time

Table 22 Change in participant satisfaction over time

| **Session Year** | **0 Stars** | | **1 Star** | | **2 Stars** | | **3 Stars** | | **4 Stars** | | **5 Stars** | |
| --- | --- | --- | --- | --- | --- | --- | --- | --- | --- | --- | --- | --- |
|  | **Rating** | **%** | **Rating** | **%** | **Rating** | **%** | **Rating** | **%** | **Rating** | **%** | **Rating** | **%** |
| **2021** | 25 | 8.9 | 27 | 9.6 | 133 | 47.3 | 67 | 23.8 | 28 | 9.7 | 1 | 0.4 |
| **2022** | 377 | 9.2 | 197 | 4.8 | 319 | 7.7 | 445 | 10.8 | 827 | 20.1 | 1954 | 47.4 |
| **2023** | 211 | 0.8 | 218 | 0.8 | 303 | 1.1 | 1844 | 6.8 | 5750 | 21.2 | 18796 | 69.3 |

### 2.3.3 Perceived Benefit


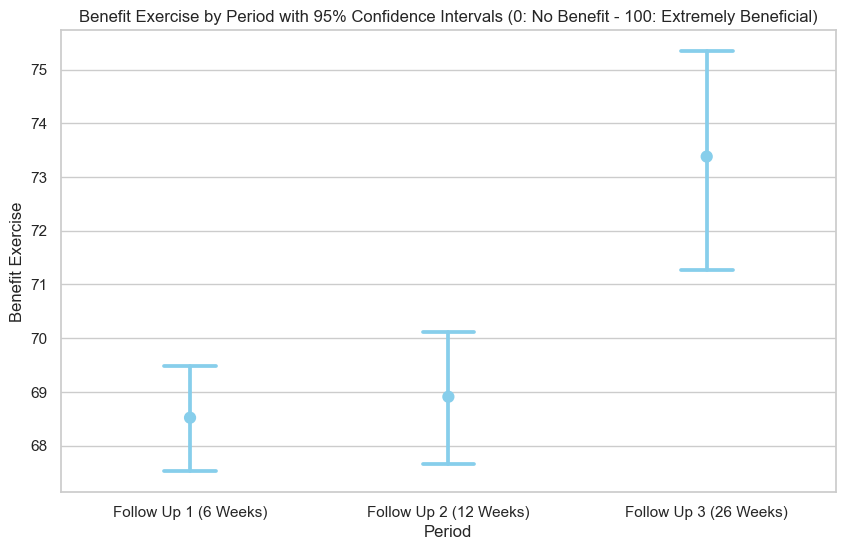


Figure 21 The perceived benefit participant felt for their primary condition over time

Table 23 Raw values for perceived benefit felt by the participants 0 = no benefit

| Time period | n | Mean | 95% CI Lower | 95% CI Upper |
| --- | --- | --- | --- | --- |
| 6 Weeks | 2087 | 68.5 | 67.6 | 69.5 |
| 12 Weeks | 1177 | 69.0 | 67.6 | 70.2 |
| 26 Weeks | 413 | 73.4 | 71.3 | 75.4 |

1. https://data.cdrc.ac.uk/dataset/index-multiple-deprivation-imd [↑](#footnote-ref-1)
